# The PluriNetWork: An Electronic Representation of the Network Underlying Pluripotency in Mouse, and Its Applications

**DOI:** 10.1371/journal.pone.0015165

**Published:** 2010-12-10

**Authors:** Anup Som, Clemens Harder, Boris Greber, Marcin Siatkowski, Yogesh Paudel, Gregor Warsow, Clemens Cap, Hans Schöler, Georg Fuellen

**Affiliations:** 1 Institute for Biostatistics and Informatics in Medicine and Ageing Research, University of Rostock, Rostock, Germany; 2 Department of Cell and Developmental Biology, Max Planck Institute for Molecular Biomedicine, Münster, Germany; 3 DZNE, German Center for Neurodegenerative Diseases, Rostock, Germany; 4 Institute for Anatomy and Cell Biology, Ernst Moritz Arndt University Greifswald, Greifswald, Germany; 5 Department of Mathematics and Informatics, Ernst Moritz Arndt University Greifswald, Greifswald, Germany; 6 Department of Computer Science, University of Rostock, Rostock, Germany; 7 Medical Faculty, University of Münster, Münster, Germany; University of Leuven, Belgium

## Abstract

**Background:**

Analysis of the mechanisms underlying pluripotency and reprogramming would benefit substantially from easy access to an electronic network of genes, proteins and mechanisms. Moreover, interpreting gene expression data needs to move beyond just the identification of the up-/downregulation of key genes and of overrepresented processes and pathways, towards clarifying the essential effects of the experiment in molecular terms.

**Methodology/Principal Findings:**

We have assembled a network of 574 molecular interactions, stimulations and inhibitions, based on a collection of research data from 177 publications until June 2010, involving 274 mouse genes/proteins, all in a standard electronic format, enabling analyses by readily available software such as Cytoscape and its plugins. The network includes the core circuit of Oct4 (Pou5f1), Sox2 and Nanog, its periphery (such as Stat3, Klf4, Esrrb, and c-Myc), connections to upstream signaling pathways (such as Activin, WNT, FGF, BMP, Insulin, Notch and LIF), and epigenetic regulators as well as some other relevant genes/proteins, such as proteins involved in nuclear import/export. We describe the general properties of the network, as well as a Gene Ontology analysis of the genes included. We use several expression data sets to condense the network to a set of network links that are affected in the course of an experiment, yielding hypotheses about the underlying mechanisms.

**Conclusions/Significance:**

We have initiated an electronic data repository that will be useful to understand pluripotency and to facilitate the interpretation of high-throughput data. To keep up with the growth of knowledge on the fundamental processes of pluripotency and reprogramming, we suggest to combine Wiki and social networking software towards a community curation system that is easy to use and flexible, and tailored to provide a benefit for the scientist, and to improve communication and exchange of research results. A *PluriNetWork* tutorial is available at http://www.ibima.med.uni-rostock.de/IBIMA/PluriNetWork/.

## Introduction

The large amount of molecular data and publications on pluripotency, reprogramming and the mechanisms underlying these phenomena, is constantly, and at times exponentially, increasing. Every month, several hundred papers are published on these topics. The in-vitro induction of pluripotency in differentiated cells by defined factors, the re-differentiation of iPS cells into various cell types, and the steady advances in refining and extending the experimental approaches transformed the field (see [1], [2] for recent reviews). While only a few defined factors can trigger induction of pluripotency, the underlying mechanisms are complex, including the up/downregulation of transcription factors, a wide array of epigenetic changes, protein post-translational modifications, effects mediated by micro-RNAs, and adaptations in cellular signaling and cell-to-cell communication. The mechanisms encompass the entire cell (nucleus, cytoplasm, membrane, …). They are dependent on space (e.g. cellular component), time (e.g. along a developmental timeline) and the cellular environment. They are cell-line specific as well as species specific to a varying degree. Moreover, the associated measurements of cellular components are subject to experimental noise and biological variability. Thus, network-based data integration efforts are inevitably resulting in an artifact:

1. Network diagrams attempt to display processes that do neither occur at the same time, nor on the same time scale, nor at the same place, nor for the same cell line.
2. At best, they have a high coverage of the most relevant relationships between cellular components, with a high percentage of correct mechanistic inferences and a low level of omission and error introduced by curation or text mining, inspector bias, and experimental error. (At worst, they are a worthless assortment of false positives.)
3. Any network representation of biological processes suffers from the inherent limitations of the representation itself: just using nodes and edges of a limited number of types over-simplifies the complicated interplay of known (and unknown) biological processes that underlie a phenomenon such as cellular pluripotency.
4. Perhaps most significantly, “pluripotency” is an ambigous term. Taking pluripotency as the state of a cell which is able to differentiate into all cell types of the adult organism, we note that this definition does not describe a directly observable fact, but the disposition, or potential, of an entity. Depending on the cellular environment and on the test of this potential (which may be in vivo, or in vitro), and on the stringency of the test, the term “pluripotency” describes a wide variety of natural as well as man-made (in vitro) cell states. If we do *not* distinguish these, our network describes an artifactual assembly of knowledge about a variety of related cellular states that are loosely described as being “pluripotent”.

With the aforementioned caveats, we nevertheless believe that an electronic representation of pluripotency is useful in principle, improving our understanding and accelerating progress via improved abilities of data analysis, generation of hypotheses and gain of insight. Therefore, we assembled the *PluriNetWork* as an interaction/regulation network describing the molecular mechanisms underlying pluripotency. Node annotations (e.g. various gene/protein identifiers) and link annotations (e.g. pointers to the literature) enable easy exploration of the network. Moreover, it can be subjected to automated analyses, yielding Gene Ontology enrichment, network statistics, and much more. Continued maintenance of the network is extremely important. Therefore, the publication-based network presented here will be placed in the WikiPathways [3] repository. Moreover, we will continue maintaining the network ourselves, and we wish to add further aspects as outlined in the conclusions. In this paper, we will describe how the network was assembled, we will describe its layout and general properties, we will describe how it may be used, and we will discuss issues of data quality and continued maintenance.

### Related Work

In the *Results and Discussion* section, we will compare our network to the network by Xu et al. [4], [5], which (to our knowledge) is the most recent and up-to-date network that is also based on a literature-curation effort in mouse. While there is a lot of overlap between both networks, we note that our network includes twice as many genes, and almost two and a half times as many links. Other types of pluripotency network are based on machine learning, using high-throughput interaction and gene expression data as input. In particular, Müller et al. [6] developed the *Plurinet*, an undirected network describing stem cell regulation in human. Starting from a background network of interaction and regulation links, including the data of [7], they used a variant of the *MATISSE* machine learning algorithm [8] in order to exploit gene expression data for an extension of the network, yielding a network of 299 genes. Following up on Müller et al., Newman and Cooper [9] used their AutoSOME machine learning (clustering) approach to generate the PluriUp gene set as a cluster of 3421 genes “upregulated in pluripotent stem cells”, and the PluriPlus network as the subset of 1165 PluriUp genes that are interconnected by verified protein-protein interactions from the Human Protein Reference Database (HPRD, [10]). Their network contains about 6% of the genes in the human genome, but it is unclear how many false positive and false negative genes and links are included/omitted, since gene upregulation, and HRPD-based interaction in any kind of experimental setting, may not bear relevance to the mechanisms directly involved in pluripotency. As will be described in more detail towards the end of the paper, manual data curation has distinctive advantages (and disadvantages), which will prompt us to suggest an approach inspired by Wiki and social networking software, towards enabling manual curation at a larger scale, for maintaining and updating the *PluriNetWork* without resorting to machine learning or text mining. A thorough discussion of the advantages, disadvantages and pitfalls of manual curation will be given towards the end of the article, in the section “*A global overview of the information flow in pluripotency – a community effort?*”.

## Materials and Methods

### Network assembly

**Figure 1:**
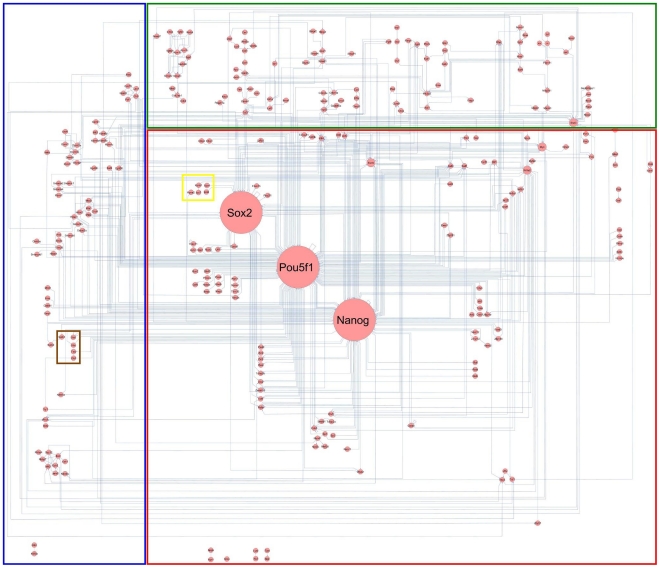
Manual layout of the *PluriNetWork* in Cytoscape. Nodes are genes/proteins, edges are stimulations (arrows), inhibitions (T-bar arrows) and interactions (lines). The top third of the network includes upstream signaling pathways, the middle is composed of the core circuitry of pluripotency (Pou5f1 (also known as Oct4), Sox2 and Nanog) and its periphery, and the left part includes epigenetic factors and related mechanisms. At the bottom, we positioned a few small subnetworks such as the interaction of Atrx with Histone H3.3 based on [64], which are not (yet) connected to the rest of the *PluriNetWork*.

Starting with the review of Sun et al. [11], we created our network (Figure 1) manually by adding nodes (genes/proteins) and edges (stimulations, inhibitions and interactions) describing *direct* mechanisms reported in the literature to have an *influence on pluripotency* in the *mouse* model system. To create an initial network, we used 14 reviews [1], [2], [11]–[22] believed to be authorative. These reviews contributed core mechanisms known before 2006. Relevant original literature was then obtained by following citations using Google Scholar, and by inspecting ‘Related Articles’ indicated by Pubmed. All additions were done manually, and are thereby subjective. The advantages and disadvantages of this approach have been discussed e.g. by Bureeva et al. [23] and we will discuss them towards the end of the paper.

The criteria for adding data to the network were set as follows:

1. *The mechanism must be described as direct.*
2. *The mechanism must be involved in the induction, maintenance or loss of pluripotency.*
3. *The mechanism must be described for the mouse model system.*

Criterion (3) is objective, even though for the few papers describing mouse data and data from other species, we had to disentangle these. Criteria (1) and (2) are subjective, and we had to be pragmatic in their application. In particular, these criteria were interpreted the stricter, the larger the amount of data under consideration. Thus, small-scale studies are given an explicit advantage simply because we posit that they report mechanisms studied in detail, so that we have more confidence that these mechanisms are both direct ***and*** relevant to pluripotency. Moreover, for researchers using our *PluriNetWork*, the references underlying the links in the network are more informative if they refer to small-scale studies. In turn, large-scale studies are given an explicit disadvantage. Of course, this rule-of-thumb also has the benefit of keeping our task more manageable. We explain each criterion in detail.

*(1) The mechanism must be described as direct.*

We distinguish three direct mechanisms called *interaction*, *stimulation* and *inhibition*. We do not distinguish a gene and its protein product, both represented by a single node in the network. The direct mechanisms give rise to direct links between the nodes. The resulting ‘binary network’ [24] closely resembles the kind of network usually displayed in reviews, and it strikes a balance between the least informative approach (that is, a network of undirected links, which may refer to any kind of direct mechanism), and more informative approaches. More informative networks may include more details about the links; our network is open to this kind of amendment, by adding further annotations to the links (and nodes), supplementing the current set of attributes (see Table 1 and Table 2). More informative networks may also include information that cannot be represented by linking genes/proteins directly. We do not include such information, which may describe reactions between more than two partners, and logical relationships (such as ‘*AND*’ and *‘XOR’*) that naturally involve more than two ‘partners’ (for example, where both A *AND* B drive the expression of C). The benefit of this exclusion is threefold: (a) it keeps our task manageable, (b) it keeps the network (which is large anyway, see Figure 1) accessible to human inspection, and (c) it keeps the network accessible to automated analyses that work with such a simple network. The downside is of course a lack of detail that may impede many kinds of analyses and insights.

**Table 1:** Attributes of selected nodes in the *PluriNetWork*.

MGI Symbol	Full name	EntrezGene ID	Unigene ID	Ensembl ID	UniProt
Sall1	sal-like 1	58198	Mm.214361	ENSMUSG00000031665	Q9ER74
Sall3	sal-like 3	20689	Mm.215917	ENSMUSG00000024565	Q62255
Sall4	sal-like 4	99377	Mm.389368	ENSMUSG00000027547	Q6S7E9

For each node (gene/protein), several identifiers are listed, using the Mouse Genome Informatics [37] symbol as the standard symbol.

**Table 2:** Attributes of selected edges in the *PluriNetWork*.

Node 1	Node 2	Interactiontype	Reference	Pubmed ID	Added by	Added on
Sall4	Mta2	interaction	Lu J, Jeong HW, Kong N, Yang Y, …	19440552	CH	2010.05.18
Sall4	Pou5f1	stimulation	Zhang J, Tam WL, Tong GQ, Wu Q, …	16980957	AS	2009.07.27
Sall4	Sall1	inhibition	Lu J, Jeong HW, Kong N, Yang Y, …	19440552	CH	2009.08.27

For each edge (link), the source and target gene/protein are listed, the interactiontype, the reference(s) incl. Pubmed IDs, and curator information.

We set specific rules for adding each of the three types of direct mechanisms:

- A *stimulation* link was added, if a direct mechanism exists, and a change in the amount/activity of the stimulator was reported to result in correlated changes of the amount/activity of the target.
- An *inhibition* link was integrated, if a direct mechanism exists, so that a change in the amount/activity of the inhibitor was reported to result in anti-correlated changes of the amount/activity of the target.
- An *interaction* link was included, if a direct mechanism exists, but the link is neither known to be a stimulation, nor an inhibition.

We derived link information from publications, manually analyzing their text, figures and tables. For example, text terms such as ‘interacts with’, ‘binds to’ or ‘forms a complex with’ usually led to inclusion of an interaction link. Text terms such as ‘stimulates’ or ‘activates’ usually led to inclusion of a stimulation link. Text terms such as ‘inhibits’ or ‘represses’ or ‘marks for degradation’ usually led to inclusion of an inhibition link. However, most stimulations and inhibitions that we included are transcriptional, that is, the stimulator/inhibitor binds directly to the regulatory region of the target; we consider this mechanism as direct, since we do not distinguish a gene and its protein product. Under this assumption, we can say that a direct physical interaction underlies all links. More specifically, the text terms ‘activates’, ‘stimulates’, ‘inhibits’ and ‘represses’ led to inclusion, when the term included the adverb ‘directly’; otherwise they were investigated further. In case of transcriptional regulation we only considered as sufficient the concurrent evidence of (a) binding to the regulatory region of the target gene by the source protein (shown, e.g., by ChIP data), and (b) the demonstration of a regulatory effect (e.g. by expression data). Promoter binding as the only evidence for a mechanism was not sufficient and therefore not considered.

Data from figures or tables are usually reporting the results of large-scale (high-throughput) experiments. As described, in these cases we were restrictive in our inclusion criteria. For large-scale data, a necessary condition for inclusion was our ability to validate the underlying evidence based on experiments described in the text. Single-step affinity purification methods tend to result in a high amount of nonspecific bindings, leading to subsequent identification of false positives, e.g. by mass spectrometry [25]. Therefore we did not integrate results obtained from single-step affinity purification. Pardo et al. [26] used tandem affinity purification, which reduces the amount nonspecific bindings [25]. We included interactions obtained by such advanced purification strategies using up-to-date equipment, believing them to be qualitatively close to small scale experiments. Further examples of included data are interactions obtained from co-immunoprecipitation experiments indicating direct associations of an antibody target with other proteins, which are subsequently identified by mass spectrometry or immunoblotting (e.g. western blotting). We note that most antibody-based data carry the false positive risk of identifying indirect relationships, because the antibody may pick a protein B that strongly interacts with another protein A, and the identification of protein A then pretends a direct relationship, which in reality is indirect, mediated by protein B.

Many interaction links are describing protein complexes. Small protein complexes such as the Il6st/Lifr complex are displayed by including interactions between each constituent protein. Problems arise when a protein P is noted to interact with the protein complex as a whole, but not with a specific part of it. In this case we added links to every protein of the complex, in case of a small protein complex, assuming that the interaction does indeed happen with all (or at least most) constituents of the complex. An example is the link from the Il6st/Lifr complex to Ptpn11 [17]. On the other hand, if the complex has a lot of constituent parts, the number of additional interactions would be inflated if we added all of them. Moreover, for big complexes such as the NuRD complex, it is usually not plausible that a protein P interacts with all constituents of the complex. Therefore, statements that a protein interacts with a large complex were not used to add links to our network.

*(2) The mechanism must be involved in the induction, maintenance or loss of pluripotency.*

Experiments promoting, attenuating or maintaining a pluripotent phenotype were considered when they reveal mechanistic data. As discussed in the introduction, we do not distinguish the various types of pluripotency (developmental, in-vitro, induced, …). We included data on the mechanisms of differentiation of pluripotent cells (embryonic stem cells or epiblast stem cells) into various lineages, as long as the mechanisms were describing the loss of pluripotency and not the gain of lineage-specific traits. We also included data on the mechanisms behind induction of pluripotency, as long as the mechanisms were concerned with the gain of pluripotency, and not the loss of lineage-specific traits. Genes involved in the cell cycle, DNA repair & DNA replication (such as cyclins and cyclin-dependent kinases) and genes involved in general epigenetic phenomena (such as histones) were only considered, if they were reported to have a specific role in pluripotency; otherwise the network would be inflated by data that are not specific to pluripotency. Along the same lines, a few links belonging to canonical pathways were added to the network, if the pathways were reported to be directly involved in pluripotency. For example, the canonical Wnt pathway is included because its relevance was already noted in the reviews we started with. From the KEGG [27] pathway ‘MAPK signaling pathway - Mus musculus’, stimulation links from Mapk1 (also known as ERK1) and Mapk2 (also known as ERK2) to c-Myc were included, because this pathway has been shown to have a strong relevance for pluripotency [28] and Mapk signalling upregulates c-Myc [29]. The Insulin/IGF signalling pathway was taken from [30], because its stimulation maintains the typical morphology of pluripotent embryonic stem cells [31].

*(3) The mechanism must be described for the mouse model system.*

Mechanisms must be described in mouse cell lines; we did not include data from any other species including human. We did not include heterokaryon data such as the data from [32]. Also, we did not distinguish between specific embryonic stem cell lines such as D3, E14, etc, or iPS cell lines such as 1D4 [33].

### Continuous network maintenance

Starting in May 2009, we set up weekly NCBI searches for relevant new publications, and filtered the resulting lists of titles for relevance. Abstracts were scanned, and papers describing mechanisms as described above were used to expand the coverage of the network.

### Network layout and functionality

**Figure 2:**
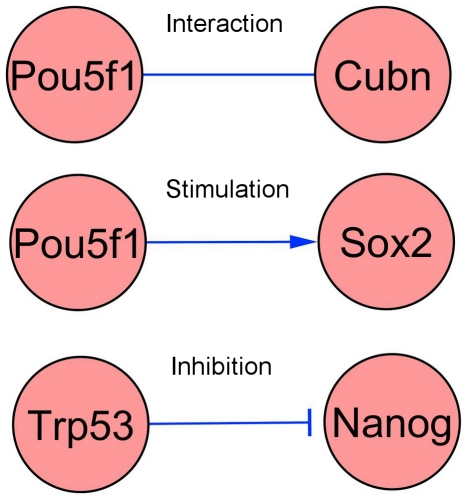
Graphical symbols of the various link types.

We aimed at a compromise between a pleasing layout guided by the idea of a ‘circuit’ representing mechanistic knowledge, and amenability to automated analysis. The network layout was produced by manual use of the Cytoscape editor [34]. Each node represents a gene and its corresponding protein product. As stated, we intentionally focused on information flow, neglecting reactions, metabolites, intracellular movement of components, and their modifications such as protein phosphorylation, and considered three types of mechanisms corresponding to three different link types. *Stimulations* are indicated by an arrow denoting the regulatory direction. *Inhibitions* are marked by a T-bar arrow. *Interactions* are displayed as simple lines (Figure 2). Terminology and graphical symbols follow Systems Biology Ontology (SBO) terms [35] and the activity flow language of the Systems Biology Graphical Notation (SBGN, [36]), where stimulation corresponds to SBO:0000170, *inhibition* to SBO:0000169 and *interaction* to SBO:0000231.

The overall layout is a “circuit” that allows easy human interpretation (Figure 1). We distinguish five regions of the network. The core region consisting of the main genes involved in pluripotency (Oct4, Sox2, Nanog, Klf4, …) and further transcription factors are placed in the center (red region). Upstream signaling pathways are located in the top third of the network (green region). Epigenetic factors are in the left part (blue region). Two small functional clusters represent proteins involved in import and export (yellow region), and X-chromosome inactivation (brown region).

Each gene/protein has the attributes described in Table 1. In particular, we provide identifiers from MGI (Mouse Genome Informatics [37]), EntrezGene [38], Unigene [38], Ensembl [39] and Uniprot [40] for each gene/protein. The labels of the nodes in the network are the symbols taken from MGI. They can be searched using the Cytoscape Search box, configuring the *node.label* as search attribute. The link attributes (Table 2) include source and target gene symbol, the type of mechanism (interaction, simulation or inhibition) and the reference. For some links, multiple supporting publications are listed. Publications are represented by their Pubmed ID (a click on the ID starts a web browser display of the abstract, provided that this functionality is enabled within Cytoscape), and directly by a text entry listing authors, title and other bibliographical information. For each entry, network curator information is recorded, including submitter and submission date.

## Results and Discussion

**Figure 3:**
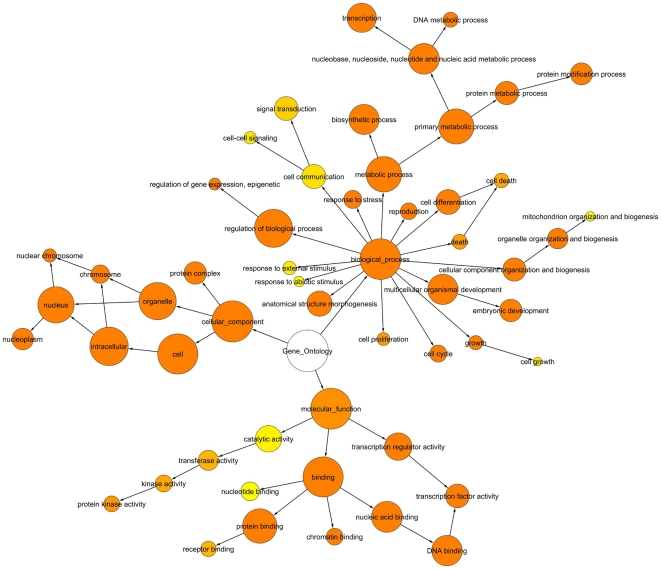
Enrichment analysis of the *PluriNetWork* genes at a significance level *p  = 0.05*, using GO terms from GO Slim. The BiNGO [43] graph visualizes the GO categories that were found significantly over-represented in the context of the GO hierarchy. According to BINGO documentation, the size (area) of the nodes is proportional to the number of genes in our gene set which are annotated to that node. The color of the node represents the (corrected) p-value. White nodes are not significantly over-represented, the other ones are (hypergeometric test, Benjamini & Hochberg False Discovery Rate (FDR) correction), with a color scale ranging from yellow (*p-value*  =  significance level, here *0.05*) to dark orange (*p-value  = 5* orders of magnitude smaller than significance level, here *0.0000005*). The color saturates at dark orange for p-values which are more than *5* orders of magnitude smaller than the chosen significance level.

The properties of the network and its components will be reported and discussed based mostly on the results of Cytoscape plugins analyzing network topology and gene ontology of the genes. Applications of the network will then be demonstrated by a set of examples. The supplementary material includes a Web tutorial (Text S1, http://www.ibima.med.uni-rostock.de/IBIMA/PluriNetWork/) and all necessary files (Data Set S1, Data Set S2) to enable the reader to reproduce the results of the following sections. In particular, the *PluriNetWork* itself is included in Data Sets S1 and S2. Figures 3–7 can be reproduced by following the detailed instructions in the Web tutorial.

**Figure 4 pone-0015165-g004:**
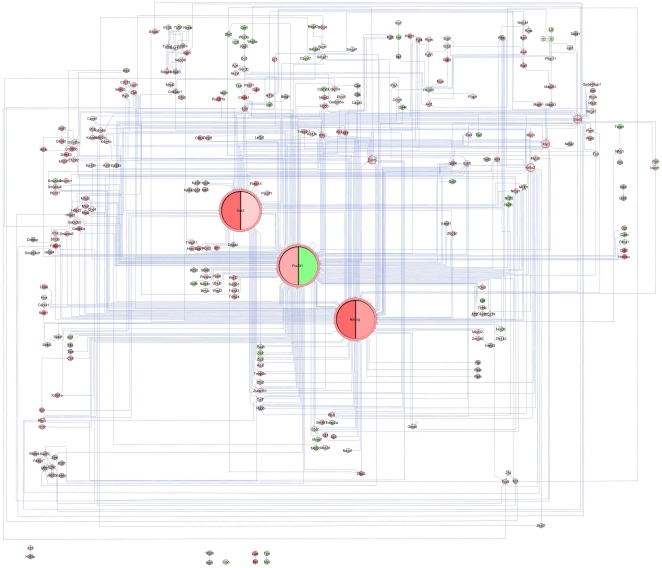
*PluriNetWork* with gene expression data, contrasting the ES cell state and day 2 of an Oct4 conditional knockout. Gene expression upregulation is denoted in red and downregulation in green. Large differences in expression yield high color intensities.

**Figure 5 pone-0015165-g005:**
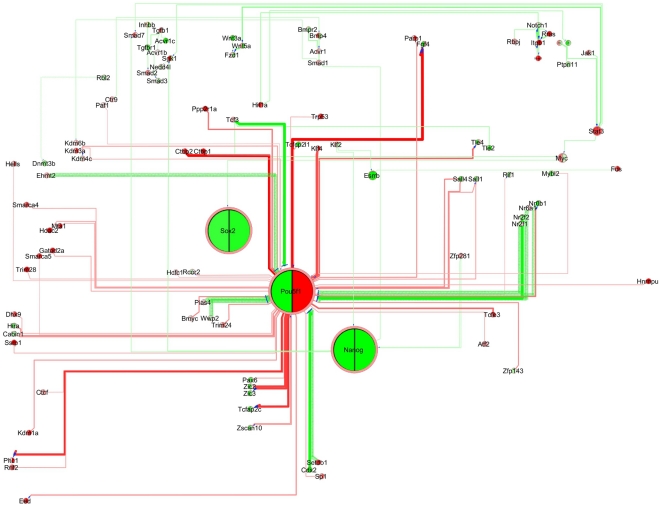
*PluriNetWork* condensed by *ExprEssence*, comparing microarray data from mouse embryonic fibroblast (MEF) and partially induced pluripotent cells (piPS). The top 10% startups (red) and the top 10% shutdowns (green) are highlighted. Link scores are based on log-transformed gene expression intensities, corrected for variance.

**Figure 6 pone-0015165-g006:**
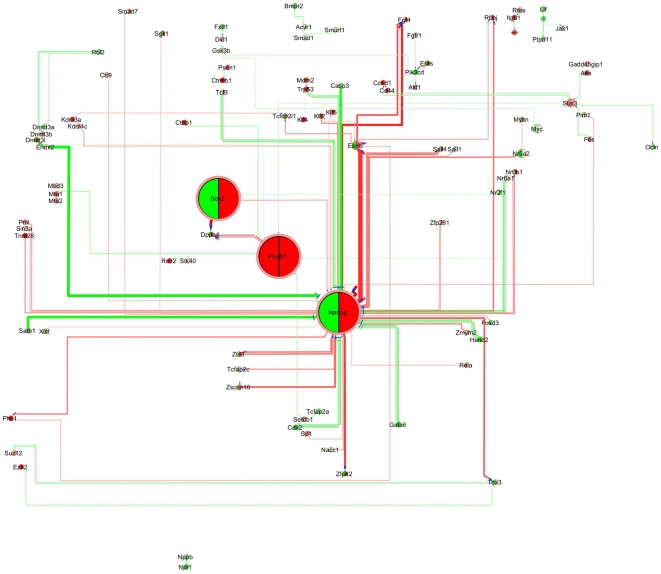
*PluriNetWork* condensed by *ExprEssence*, comparing microarray data from mouse partially induced pluripotent cells (piPS) and induced pluripotent cells (iPS). See also Figure 5.

**Figure 7:**
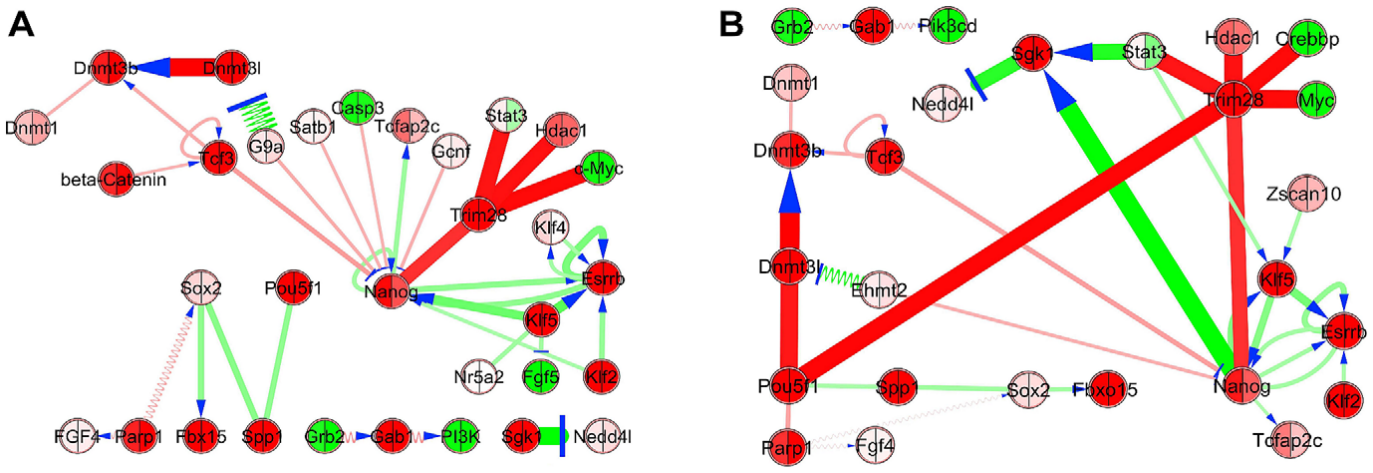
*PluriNetWork* condensed by *ExprEssence.* March 2010 version (A) and July 2010 version (B), comparing microarray data from two murine ES cell experiments: (1) “12h PD LIF” and (2) “12h PD Jaki” (see Table 3). The top 5% startups (red) and the top 5% shutdowns (green) are highlighted. Link scores are based on the original gene expression intensities. Panel A on the left is adapted from Warsow et al. [50]. The layout is done manually; the ‘circuit layout’ of the *PluriNetWork* for a condensed network including only 10% of the links would be dominated by white-space, even more than in Figure 5, which features 20% of the links.

**Table 3:** Summary of the four different treatment conditions applied to mouse embryonic stem cells in [53].

Treatment condition		LIF	LIF inhibition by Jaki
	*downstream targets*	*Stat3;* *Klf4*	inhibition of *Stat3;*inhibition of *Klf4*
**FGF inhibition by PD**	inhibition of *MEK/ERK;*inhibition of *Klf2*	(1) 12h PD LIF⇒ ES state maintained	(2) 12h PD Jaki⇒ partial transition to Epiblast
**FGF**	*MEK/ERK;* *Klf2*	(3) 12h FGF LIF⇒ partial transition to Epiblast	(4) 12h FGF Jaki⇒ transition to Epiblast

(For each intervention, its effect on the two downstream targets is listed.).

### Network topology, and Gene Ontology analysis

To examine the overall structure of the *PluriNetWork*, its topology was analyzed with *NetworkAnalyzer*, a Cytoscape plugin developed by Assenov et al. [41]. We considered our network as undirected, treating every link as an interaction link, to accommodate the input requirements to *NetworkAnalyzer*. The *PluriNetWork* consists of 274 nodes and 574 edges with an average node degree of 3.85. The network diameter is 10, the clustering coefficient 0.26 and the characteristic path length 3.25. These properties are in close correspondence with the data reported by [42]: Literature-curated networks containing interactions observed in at least one experimental study (LC-1) are expected to have an average node degree of 7.00, a diameter of 12, a clustering coefficient of 0.27 and a mean path length (which is a synonym for characteristic path length) of 4.22. Interestingly, while the last three parameters are matching best to an LC-1 network, the average node degree of our network is between the average node degrees of an LC-2 literature network (that is, 4.21) and an LC-3 literature network (that is, 3.51), indicating support by at least two to three different experimental studies. Indeed, many links in our network have multiple references that back them up.

Figure 3 describes an overrepresention analysis of the biological processes and molecular functions performed by the genes in the *PluriNetWork*, based on the Gene Ontology of all its 274 genes. We used BINGO [43] with the GO Slim Generic Gene Ontology Annotation [44], which is a set of high-level GO terms. As detailed in the Web Tutorial (http://www.ibima.med.uni-rostock.de/IBIMA/PluriNetWork/), GO terms such as “transcription” and “embryonic development” are highlighted. This is not surprising, but it can be taken as evidence that the proteins/genes of the network perform functions and biological processes related to pluripotency.

### Comparing the PluriNetWork to another literature-based network, and visualization of pluripotency-related data

We compared our network with the largest literature-based network currently available, describing pluripotency in mouse, reported recently by Xu et al. [4] (see also [5]). The network was created from the edge list downloaded at http://amp.pharm.mssm.edu/iscmid/literature/, and it consists of 134 genes/proteins (nodes) and 220 links (edges). In contrast to the *PluriNetWork*, the Xu et al. [4] network contains indirect links, e.g. as part of signalling pathways. Examples for such indirect links are: *Akt1 stimulates Tbx3, Irs1 stimulates Pou5f1, Lif stimulates Stat3,* and *Sox15 stimulates Otx2*. After removing indirect links, the intersection of our network and theirs contains 101 links, an overlap of 46%. The node overlap consists of 86 nodes (64%). Among the genes/proteins missing in our network are histones such as Hist3h3, Hist2h3c and Hist4h4. We did not include these because they are involved in general epigenetic phenomena, and do not play a specific role in pluripotency, even though they are mentioned in papers discussing pluripotency. Also missing in our network are the genes Ccrn4l, Rgs16, Spry2, Cnnm1, Dact1, Gbx2, Ier3. These are included in the Xu et al network based solely on binding of their promoters by Nanog and/or Stat3 [45]. Xu et al included some more links based solely on promoter-binding data reported by [46], and they also included links based on computational promoter-binding predictions [47]. Both lines of evidence are not sufficient for inclusion by our criteria.

In Figure 4, we visualized the loss of pluripotency in the *PluriNetWork,* identifying agonists and antagonists of this cellular state. We used the microarray data of [48], reported in [49] (GSE10477), describing the change of mouse ES cell gene expression after two days of Oct4 (Pou5f1) conditional knockout, yielding trophectodermal morphology [49]. For each gene, one pie chart describes its expression pattern, where the color of each slice is based on one gene expression value (left: gene expression on day 0, right: gene expression on day 2 of Oct4 knockout), rendering low expression values in green, intermediate levels in white, and high values in red. As detailed in the Web Tutorial (http://www.ibima.med.uni-rostock.de/IBIMA/PluriNetWork/), agonists and antagonists of pluripotency are highlighted. Again, this is not surprising, but it can be taken as evidence that the network is indeed strongly associated with pluripotency.

### Explorative data analysis using the ExprEssence Cytoscape Plugin

While a binary network just featuring interactions, stimulations and inhibitions may lack sufficient detail for some applications, in case of large networks it has some distinctive advantages: Computational analyses have less tendency of overfitting, and analysis results are easier to interpret by human inspection. Towards the latter, we have developed a software application called “ExprEssence”[50], which highlights the binary links across which the largest amount of change can be observed, given two experimental data sets. More specifically, *ExprEssence* condenses networks so that they contain only those links between genes/proteins, along which a large amount of change in (expression) values takes place. These links are called *most differentially altered.* The percentage of *most differentially altered* links to be highlighted can be set by the user. Highlighting identifies hypotheses about the startup or the shutdown of interactions, stimulations and inhibitions. *ExprEssence* is available as a Cytoscape [34] plugin at http://sourceforge.net/projects/expressence/. For the microarray data sets described in the following, we found that the *PluriNetWork* revealed mechanistic hypotheses that were matching expert knowledge, and/or provided predictions that could be validated, thereby providing some indirect evidence of network quality. When carrying out analyses using the *PluriNetWork*, it should be noted, however, that transcriptional regulatory interactions and protein interactions are often taking place at different time scales. As we will see, *ExprEssence* analyses not only put the network to use, but they also allow insights into the network itself.

### Transition of fibroblasts to partially induced (piPS) and induced (iPS) pluripotent stem cells

Reprogramming of somatic cells to a pluripotent state is assumed to include an array of epigenetic modifications, and a reactivation of pluripotency-associated genes [33], [51]. Recently, Sridharan et al. [33] reported gene expression data (GSE14012) on three sets of murine cells: fibroblasts (MEF, mouse embryonic fibroblasts), partially induced pluripotent stem cells (piPS) and induced pluripotent stem cells (iPS), identifying characteristic transcription factor binding and gene expression patterns for these cell types and formulating hypotheses about the transitory events from fibroblasts to piPS, and from piPS to iPS cells. Induction of pluripotency is attempted by viral overexpression of the ‘Yamanaka factors’[52] Oct4 (Pou5f1), Sox2, Klf4 and c-Myc. Sridharan et al. [33] concluded that reactivation of the pluripotency genes Oct4, Sox2, Klf4 drives the induction of pluripotency, where Nanog may be a key factor for *full induction.* Indeed, by *ExprEssence* condensation of the *PluriNetWork* highlighting putative mechanisms of *partial induction* (Figure 5) and of *full induction* (Figure 6), we observe Oct4/Pou5f1-driven startup of epigenetic factors during *partial induction*, and Nanog-driven startup of pluripotency-related transcription factors during *full induction*, including Esrrb, Sall4, Tbx3, Zfp42 and Zic3. A detailed analysis is provided in the Web Tutorial (http://www.ibima.med.uni-rostock.de/IBIMA/PluriNetWork/).

### Transition from the embryonic to the epiblast stem cell state

For a network aimed at fostering our understanding of pluripotency, it is of special interest to employ it for the comparison of different cell lines that share the label of being pluripotent. These may be ES (embryonic stem) cells and iPS cells, or these may be ES cells and epiblast stem cells. The latter were already investigated in an *ExprEssence* case study in Warsow et al. [50], using a March 2010 version of the *PluriNetWork* and the microarray data (GSE10017) from Greber et al. [53]. Here, we will first repeat some analyses with the newest version of the *PluriNetWork* described here, to find out how additions to the network affect the outcome of analyses of microarray data in the context of the network. We will also analyze all four data sets (experimental conditions, see Table 3) described in [53].

We start by contrasting two of these experimental conditions: (1) “12h PD LIF” and (2) “12h PD Jaki”. For these two conditions, we obtained gene expression of mouse embryonic stem cells, (1) following 12 hours of treatment with an FGF/MEK/ERK inhibitor (PD0325901, abbreviated PD) and LIF, to maintain the ES cell state, and (2) following 12 hours of treatment with PD and with an inhibitor of LIF/JAK/STAT signaling, the “JAK inhibitor I” (Jaki, Merck). FGF signaling together with inactivation of LIF/Stat3 signaling by Jak inhibition induces a transition of mouse ES cells to the epiblast stem cell state [53], while inhibition of FGF signaling by PD together with inactivation of LIF/STAT3 signaling by Jak inhibition induces a partial transition (condition (2), see Table 3). Stat3 signaling contributes to maintaining the ES cell state, in part by stimulating its target Klf4 [54]. Consequently, links from Jak to Stat3 and from Stat3 to Klf4 are incorporated in the *PluriNetWork*. FGF/MEK/ERK signaling has been revealed to have a repressive effect on Klf2 [53]. It is, however, not known whether this effect is direct or indirect and it could, therefore, not be included in our network.

We were first interested in the stability of analyses based on our network, given that new data are added on a weekly basis as part of our continuous maintenance. The *PluriNetWork* as of March 2010 consists of 261 genes and 487 links, while the *PluriNetWork* described in this paper contains 274 genes and 574 links. Contrasting conditions (1) and (2) as described in the last section, and keeping the 5% most strongly differentially altered links, we obtained condensed networks as in Figure 7, panel A (March 2010 network, used in Warsow et al. [50]) and panel B (*PluriNetWork* as described here). The condensed networks match closely, and we observe the following in both: (a) the shutdown of stimulations around the Esrrb gene; we were already able to validate full downregulation of Esrrb at 48 hours (Warsow et al. [50]), and (b) the startup of interactions around the transcriptional co-repressor TRIM28 (also known as TIF1beta); one of its repressed targets is Stat3. Novel observations enabled by recent additions to the *PluriNetWork* are: (c) stimulation of the DNA methyltransferase Dnmt3b by Pou5f1/Oct4 (via Dnmt3l, [26]) and (d) shutdown of the inhibition (by phosphorylation) of Nedd4l by the serine/threonine-protein kinase SGK1, as a result of the shutdown of the stimulation of SGK1 by Nanog and by Stat3. According to the corresponding paper [55], the effect of the shutdown of Nedd4l inhibition is the startup of its default binding of activating Smad2/3, thus limiting TGF-beta signaling [56].

**Figure 8:**
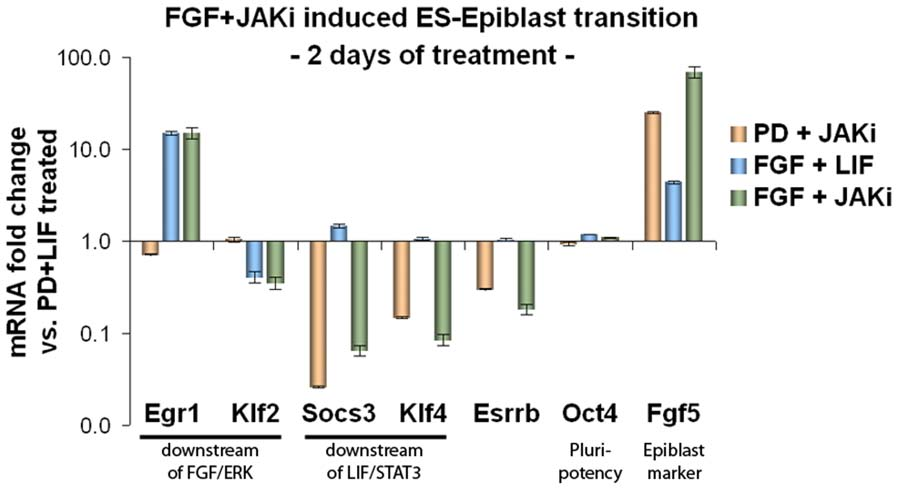
FGF stimulation and JAK inhibition promote ES-Epiblast transition. ES cells were treated for two days with activators and inhibitors of the FGF and JAK pathways, as indicated, and then subjected to quantitative real-time RT-PCR. Egr1 and Socs3 are known downstream targets of these pathways, respectively. Hence, their expression correlates well with the activation status of the two pathways, depending on the corresponding treatment conditions. Klf2 appears to be a repressed target of FGF/ERK signaling, whereas Klf4 is downstream of LIF/STAT3. Note the cooperation of FGF/ERK activation and LIF/STAT3 repression by Jaki in diminishing ES cell-specific Esrrb and in activating epiblast-specific FGF5 (data are in logarithmic scale). Notably, Oct4 levels were preserved, in line with the fact that it is expressed both in ES and epiblast stem cells.

A detailed analysis is provided in the Web Tutorial (http://www.ibima.med.uni-rostock.de/IBIMA/PluriNetWork/). It includes a discussion of two further conditions, (3) “12h FGF LIF”, and (4) “12h FGF Jaki”, see Table 3. In summary, we observe shutdowns around Klf4 and Esrrb in condition (2) “12h PD Jaki” and condition (4) “12h FGF Jaki”, so we conclude that LIF signaling inhibition by Jaki acts via Klf4, in concordance with Table 3 and confirming experimental data [54]. We observe shutdowns around Klf2 in conditions (3) “12h FGF LIF” and (4) “12h FGF Jaki”, so we conclude that FGF acts via Klf2, once more in concordance with Table 3 and confirming experimental data [53]. Finally, we found that expression of these genes (Klf4, Esrrb, Klf2) diminishes after 48 hours, in a pattern as expected, see Figure 8.

### Future work

Towards an electronic representation of the mechanisms underlying pluripotency, we believe that our manually curated network of interaction and regulation is a good starting point. For once, our network reflects the kind of information presented in reviews. Secondly, it can nevertheless be subjected to automated analyses as described in this paper. Inclusion of data on regulatory RNA (such as microRNAs, [57]) is on our agenda. The most significant shortcoming is the missing distinction between various types of pluripotency, and we have started to include link annotations to distinguish these. Once this annotation is complete, the user of the network can filter links based on the annotation, e.g. restricting an analysis to knowledge obtained about developmental in-vivo pluripotency. We have also started adding small molecules to the *PluriNetWork* (data not shown), as stimulators or inhibitors of specific genes/proteins, wherever such mechanistic data are available. We believe that such information may help to identify small molecules with an effect at the earliest time points of development, or of induction of pluripotency. For example, small molecules affecting the highlighted startups and shutdowns hypothesized for the transitions from fibroblasts to partially induced pluripotent cells, and further from these to fully induced iPS cells (see section *Transition from fibroblast to partially induced (piPS) and induced (iPS) pluripotent stem cells*), may accelerate reprogramming. More generally, we envision to integrate entire time series of expression data tracking a developmental process, or an induction process, into the network, and to create “movies” highlighting putative mechanisms in time. Time-dependent interventions may then be suggested based on these, towards supporting reprogramming or cell differentiation in a step-by-step manner.

### A global overview of the information flow in pluripotency – a community effort?

Assembly of the *PluriNetWork* as described here turned out to be a challenging exercise, because of the large and ever-growing amount of data to be curated. Nevertheless, we see a lot of value in hand-curated network data, which is complementary to networks based on machine learning or text mining. As pointed out by Bureevas et al [23], an advantage of manual curation is accuracy. Since there exist no standards for reporting an interaction or a regulation link in a paper, a human curator is best suited to understand the precise semantics of the textual descriptions provided by authors. In some articles, species and/or experimental procedures are mixed. Usually, a curator can disentangle the results reported, based on context, and interpret tables, figures and figure legends correctly.

Then again, human cognition is prone to error, because there is a tendency to simplify observations, and to propose explanations (narratives) that ignore the inherent complexity of biological phenomena. What is even more, curation may add another simplification step, subsequent to the simplifications by the authors of the original papers. The main guard against this ‘subsequent simplification’ is careful reading of the original literature (which we did), and awareness of the ‘simplification’ problem when interpreting results of analyses based on the *PluriNetWork* (something we must ask the users of our network to do).

Another disadvantage of human curation is the time it consumes, and since the number of curators is limited, their ability to catch up with the growing number of publications is limited as well. We suggest that this limitation may be overcome by a Wiki-based community effort.

Such an effort must be as open as possible, guarding against self-perpetrating misconceptions and false beliefs. In a fast-paced field such as stem cell research, there may be a particular tendency to ‘follow the crowd’, creating undue inspector bias and even false beliefs. However, the knowledge we curated does not encompass anything what may be called a ‘scientific theory’; we merely accumulate ‘small observations’ that may eventually yield a ‘theory of pluripotency’.

Thus, we will contribute the *PluriNetWork* to WikiPathways [3], which allows the upload of networks in ‘binary format’. In particular, there is limited support for import and export of the *PluriNetWork* using the Cytoscape GPML plugin. However, a network as large and rich as the *PluriNetWork* is not trivial to contribute and maintain without additional tools and features:

- A Wiki-based effort should preserve all (or at least most) attributes attached to nodes and links in the *PluriNetWork*. Currently, these must be reorganized and forced to fit into the WikiPathways representation.
- The ‘circuit layout’ of the *PluriNetWork* should be preserved. At the time of writing it can only be transferred partially to WikiPathways: the position of nodes can be conserved, but the exact course of the links connecting the nodes cannot.
- Network editing using WikiPathways is currently not as easy as it could be, e.g. we found it difficult to locate a gene/protein to which a link shall be added.
- The contributor reward system at WikiPathways may not be sufficient to attract a critical mass of contributors.
- An optimal balance between tight quality control on the information created and well understood by a single author or research group on the one hand, and the benefits of keeping the system open to amendments, corrections and annotations of other researchers on the other hand, must be found. With the possibility of unauthorized user account registration, data must be guarded against blatant vandalism and subtle, but incorrect modifications.

The last issue is a core conflict of any open Wiki-like knowledge management system. To resolve it, we suggest to combine the WikiPathways “central resource” approach with a “personalized resource” approach, where every user has her/his private copy (or variant) of the data, and to manage trust using a “social networking” approach [58], [59], where being part of a network of “friends” and repeated interaction with others increases the readiness to accept changes of others into one's own private copy.

More specifically, we suggest the following scenario for *PluriNetWork* distribution and maintenance, once it is available at WikiPathways. Starting with the *PluriNetWork* and its (updated) versions on the WikiPathways website, a scientist can “import” her/his “trusted *PluriNetWork*” as follows, with minimum effort.

1. She/he defines a version of the network as a personal variant at her/his exclusive disposal. This can be implemented either by creating a copy from WikiPathways, stored locally on the notebook of the researcher (“localized architecture”), or by creating a user page at WikiPathways, to which updated network information is attached, and access is restricted for other users as desired (“trusted architecture”).
2. In both “architectures” she/he provides edit (or just viewing) permissions to a set of “trusted curators”, taken from a list of all curators. At the moment of writing this manuscript, curators of the *PluriNetWork* can be identified for each link between genes/proteins, based on the attribute “Added by”, and they consist of the first authors of this manuscript. At some later time, curators may join at WikiPathways, and they can again be identified by the same link attribute. (These curators, as well as others, will finally be selected based on the list of all members of the entire social network of *PluriNetWork* curators, see below.)
3. In both “architectures” she/he has her/his own personal copy for editing; she/he becomes a curator him/herself, joining the “social network” of *PluriNetWork* curators. (Note that one of the partners in the “social network” may be the WikiPathways website itself.)

The benefit of this system is improved quality control by restricting access to mutually trusting curators for a network; the price to pay is the additional work of synchronizing the drift between the variants; the challenge therefore is the support of the system for automating large portions of this synchronization. The trust mechanisms of social networks propose numerous approaches, which have been positively evaluated in the context of text-only Wiki systems. An explicit trust level system can group scientists into several levels, such as:

- Scientists trusted unconditionally, to which all modifications are communicated (e.g. members of the scientist's own working group) and, similarly, from which all modifications are automatically transferred into one's own variant, and
- Scientists not trusted, for which communication of modifications must be explicitly permitted; such an explicit permission may be issued for a selected set of modifications if a certain condition is met.

For example, modifications may only be communicated to untrusted scientists upon acceptance of the publication in which they are described. In turn, the scientist will be able to define from which other scientists she/he accepts modifications of her/his network, usually with the idea of reviewing these before importing them. Software support is needed for this review; if links are just added, including them into a given network implies creation of the union of the links already there and the links to be added. However, if modifications are done, conflicts may arise and they need to be identified and resolved. Synchronization will detect and report conflicting information. Subsequent review and conflict resolution can update the central copy and the personal copy at the same time.

Improved visualization can give clues on the trust level of individual information elements; a variant can incorporate information of different levels of trust and may be filtered accordingly by asking the system to present only those elements of a pathway whose curator exceeds a certain trust level. This is a benefit for the individual researcher as well, since incomplete or not yet fully verified information can be stored in the system. Trust levels can also be established implicitly. For example, the system can track the accept/reject decisions and map them to bonus points awarded to respective curators. A certain number of bonus points would automatically place curators into different trust levels; moreover, bonus points can form the currency of contributory rewards, ranking curators by their number of accepted/rejected modifications. Similar mechanisms have succeeded in text-based Wiki systems and forums [60], [61] and trust feedback seems possible as well [62]. The final effect of these types of architectures is a decentralized store of networks for the use of research groups. Information variants dissenting with the “official” WikiPathways site are possible as well as pre-publication or “private” variants. On the other hand, tools for merging variants into a new and regained consensus are supported. Similar to distributed software and document versioning systems [63], branches (i.e. alternative solutions to problems) can be studied and merged into a final document, once consensus has been reached.

## Supporting Information

Text S1 **Web Tutorial including further information about the** ***PluriNetWork*** **and various analyses, including analyses by the ExprEssence Cytoscape plugin (see** http://www.ibima.med.uni-rostock.de/IBIMA/PluriNetWork/for **updates).**
(DOC)

Data Set S1 **Cytoscape file needed for reproduction of panel B of** **Figure 7** **. Also includes the entire** ***PluriNetWork*** **for inspection. (S1_Epiblast.cys/.rar).**
(RAR)

Data Set S2 **Cytoscape file needed for reproduction of** **Figures 5** **+ 6. (S2_MEF_PIPS_IPS.cys/.rar).**
(RAR)
