# Supplementary material for: The PluriNetWork: An Electronic Representation of the Network Underlying Pluripotency in Mouse, and Its Applications
Source: PLoS One. 2010 Dec 10;5(12):e15165. doi: 10.1371/journal.pone.0015165 (PMC3003487; doi:10.1371/journal.pone.0015165)
Supplement: Text S1 — Web Tutorial including further information about the PluriNetWork and various analyses, including analyses by the ExprEssence Cytoscape plugin (see http://www.ibima.med.uni-rostock.de/IBIMA/PluriNetWork/for updates). (DOC) [file pone.0015165.s001.doc]

**Doc-file of the Webtutorial at:** <http://www.ibima.med.uni-rostock.de/IBIMA/PluriNetWork/>

**Tutorial: The *PluriNetWork / ExprEssence* framework**

**Contents**

- [Motivation](#Motivation)
- [The *PluriNetWork*](#The_PluriNetWork)
  - [Exploring the *PluriNetWork*](#Exploring_the_PluriNetWork)
  - [Finding a gene in the *PluriNetWork*](#Finding_a_gene)
  - [Inspecting Attribute Values](#Inspecting)
  - [Getting the underlying Pubmed article(s)](#Getting)
  - [BiNGO Gene Ontology analysis](#bingo)
- [*ExprEssence*](#ExprEssence)
  - [Installation](#Installation)
  - [General *ExprEssence* Workflow](#General_ExprEssence_Workflow)
- [Case studies](#CaseStudies)
  - [MEF to piPS and piPS to iPS](#MEF_pips_ips)
  - [Embryonic Stem Cell to Epiblast Stem Cell state](#ESC_to_Epiblast)
  - [NodeColor-](#NodeColor_v) [Oct4 knockdown data](#NodeColor_Oct4_CaseStudy)
- [References](#References)
- [About](#About)

**Motivation**

We wish to obtain insights into biological processes by building up process- and species-specific protein/gene interaction and regulation networks and combining them with differential biological data (e.g. gene expression data for two time points). Towards this aim, we built up a network describing pluripotency in mouse and used it for stem cell data analysis.

**The *PluriNetWork***

The [*PluriNetWork*](http://www.ibima.med.uni-rostock.de/IBIMA/PluriNetWork/MEF_piPS_iPS.cys), described in the [*PluriNetWork* paper](http://www.ibima.med.uni-rostock.de/IBIMA/PluriNetWork/PluriNetWork.pdf)*,* is a manually curated protein/gene interaction and regulation network with the purpose of describing pluripotency in mouse. The structure of the *PluriNetWork* is straightforward; each node represents one gene and its corresponding protein, and links are either interactions, stimulations or inhibitions. As of July 2010, the network is based upon 177 publications, consists of 274 nodes (genes/proteins) and 574 edges (links) - each representing a direct interaction or regulation between two nodes. The *PluriNetWork* is explored, maintained and analyzed using *Cytoscape (see Figure 1),* (downloadable at <http://www.cytoscape.org/download.html>).


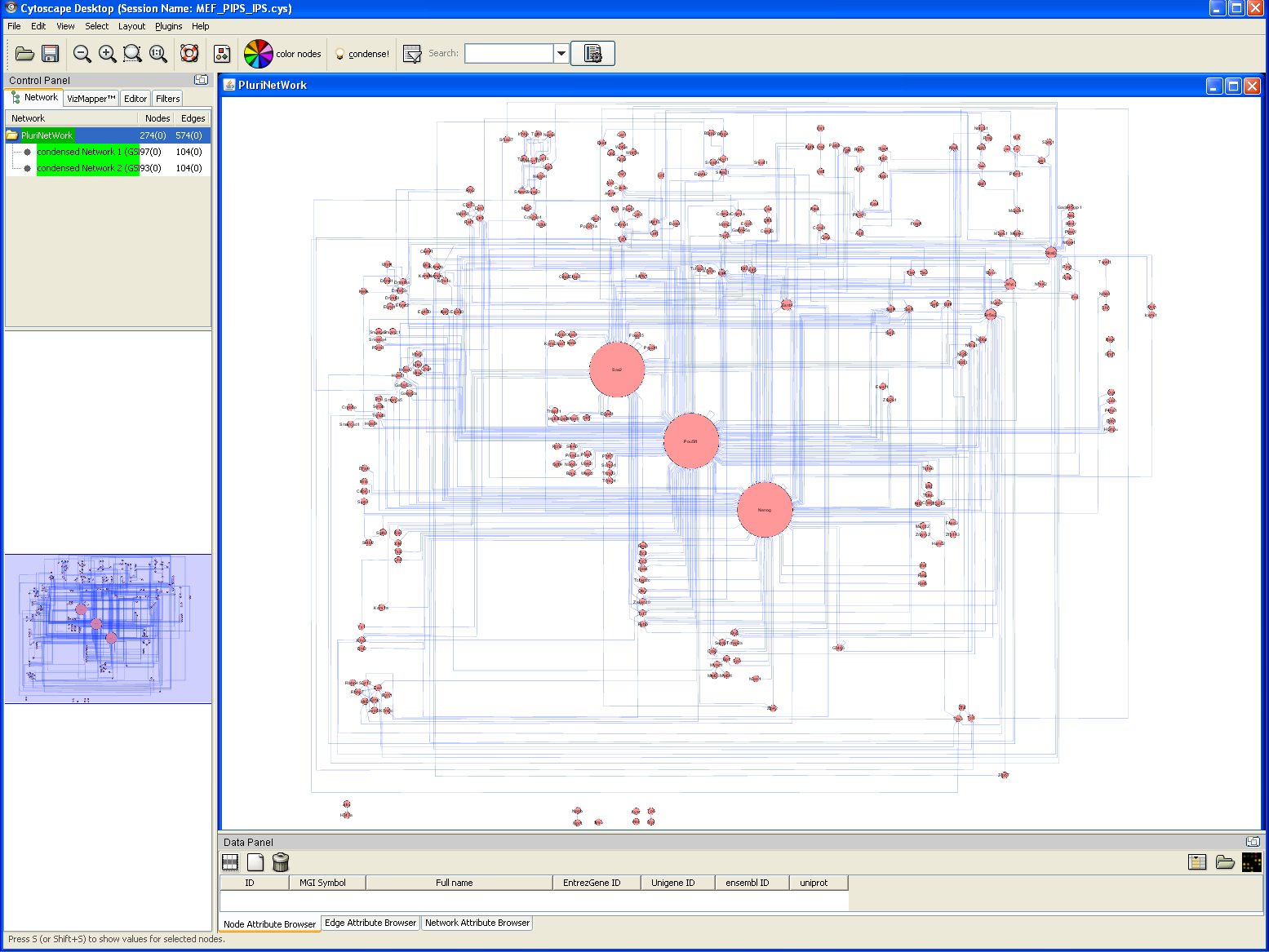


Figure 1. The *PluriNetWork*, displayed using the *Cytoscape* network visualization and data integration platform.

**Exploring the *PluriNetWork***

Apart from the circuit-like layout, the *PluriNetWork* data is represented by two tables - one table describing the nodes (genes/proteins) and their attributes and the other the edges (links) and their attributes. At any given time, only one table can be shown in the *Cytoscape* *Data Panel*. To switch between the tables, you can click at the bottom of the *Data Panel* on the tab *Node Attribute Browser* or *Edge Attribute Browser* (see Figure 2).


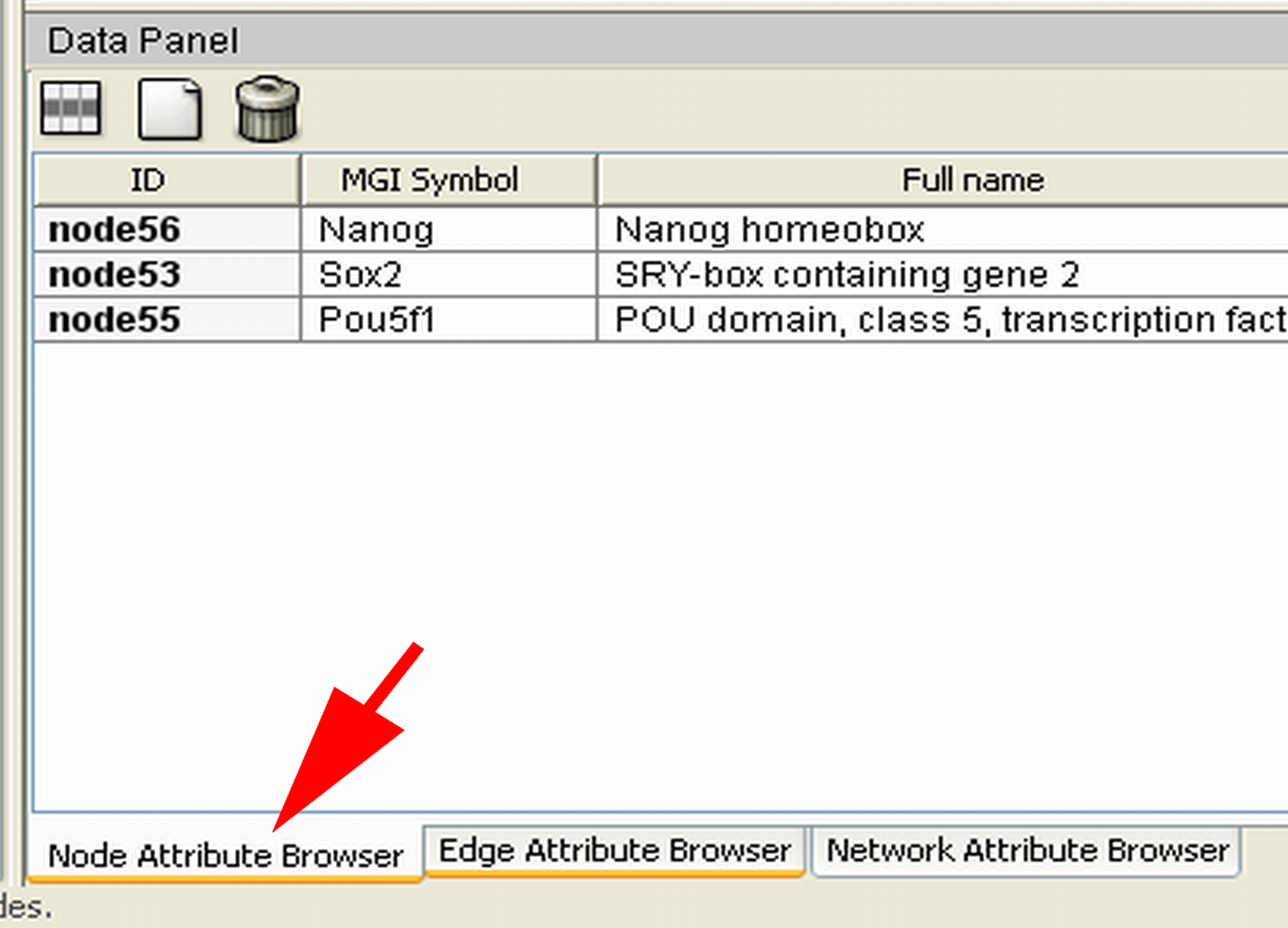


Figure 2. The *Data Panel* in *Cytoscape*.

**Finding a gene in the *PluriNetWork***

1. Click on the button ***right*** to the search input field to configure your search options (see Figure 3). The radio button that appears in the configuration dialog must be set to *Nodes*.


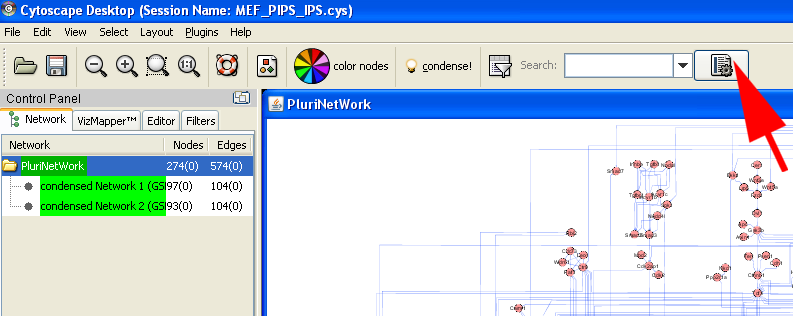


Figure 3. Starting the search configuration dialog.

1. Select an attribute containing the values you will be using for the search (e.g. an identifier such as *MGI Symbol*, to search for Pou5f1, Sox2, Nanog, Esrrb, etc).
2. Type your query into the search input field.
3. If there exists a node (gene/protein) with the attribute/value pair used for the search, this node will be moved to the center of the main panel (if the value cannot be found, the search input field will be marked by a red background).

A subnetwork can easily be retrieved in Cytoscape by selecting the desired nodes and edges and clicking on “File->New->Network->From selected nodes and edges”.

**Inspecting Attribute Values**

In the *PluriNetWork*, for each node (gene/protein) and edge (link), attributes can be browsed as follows.

1. Mark the node/edge of interest by a left mouse click (the color should change); if more than one node/edge shall be selected, hold *Shift* during selection, or select all nodes/edges in an area defined using the left mouse button.
2. Select the *Node Attribute Browser* or *Edge Attribute Browser* at the bottom of the *Data Panel*.
3. To define which attributes shall be shown, click on the first button of the *Data Panel* (see Figure 4), and then tick the corresponding checkboxes in the dialog window that opens up.
4. The selected attribute values of the selected nodes/edges are then shown in the *Data Panel*.


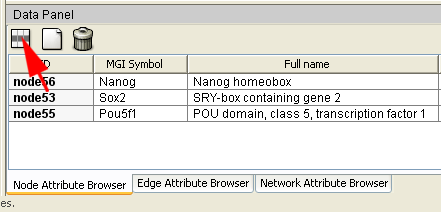


Figure 4. Starting the attribute browser.

**Getting the underlying Pubmed article(s)**

For each link, at least one reference is available in the *PluriNetWork* to back it up. It can be retrieved as follows.

1. Mark the edge for which you want to get the PubMed entry.
2. Choose the tab of the *Edge Attribute Browser* at the bottom of the *Data Panel*.
3. Left-click on the Pubmed ID attribute (if necessary, follow the section [Inspecting Attribute Values](#Inspecting), above, so that the Pubmed ID attribute is shown.)
4. The standard browser should open with the abstract of the Pubmed article. More references may be available by inspecting the attributes *Pubmed ID Source 2*, etc. (If necessary, follow the section [Inspecting Attribute Values](#Inspecting), above, so that these attributes are shown.)

**BINGO Gene Ontology analysis**

*Cytoscape* can be enhanced by a variety of plugins, allowing, for example, to perform network statistics, or to import data of different formats. For the *PluriNetWork* paper, we performed an overrepresentation analysis of the biological processes and molecular functions, based on the Gene Ontology Terms of all 274 genes. We used BiNGO [1] with the GO Slim Generic Gene Ontology Annotation [2], which is a set of high-level GO terms. The significance level was set to *p=0.05* (hypergeometric test, Benjamini & Hochberg False Discovery Rate (FDR) correction). Overrepresented terms are visualized in Figure 5 below.

To perform this analysis yourself, start *Cytoscape*, using the “*Plugins->Manage Plugins*” dialog to install the BiNGO plugin available in the *Functional Enrichment* folder, and open the file [MEF_PIPS_IPS.cys](http://www.ibima.med.uni-rostock.de/IBIMA/PluriNetWork/MEF_piPS_iPS.cys), which includes the *PluriNetWork*. Select all nodes in the network, using “*Select->Nodes->Select All nodes*” in the main *Cytoscape* menu. In the *Data Panel* of *Cytoscape*, select the *Node Attribute Browser*, and, following the instructions on [Inspecting Attribute Values](#Inspecting), select all values of the attribute *MGI Symbol* and copy the values into the clipboard. Then, start BiNGO via the Plugins menu. Define a name for your BiNGO analysis and select “*Paste Genes from Text*”. Then, paste the contents of your clipboard into the open dialog field. In the field Select “*organism/annotation”*, please select *Mus musculus*. For the analysis described here, all other parameters are default values. Start the analysis by clicking on *Start BiNGO*. A new network is generated with the name you have chosen above. This network consists of the GO Terms which could be mapped to gene names in the starting network. GO Terms which are overrepresented in your network will be highlighted. In the panel *BiNGO output*, you will find more information about the GO Terms (GO identifiers, significance of overrepresentation, names of genes mapped to this GO Term).


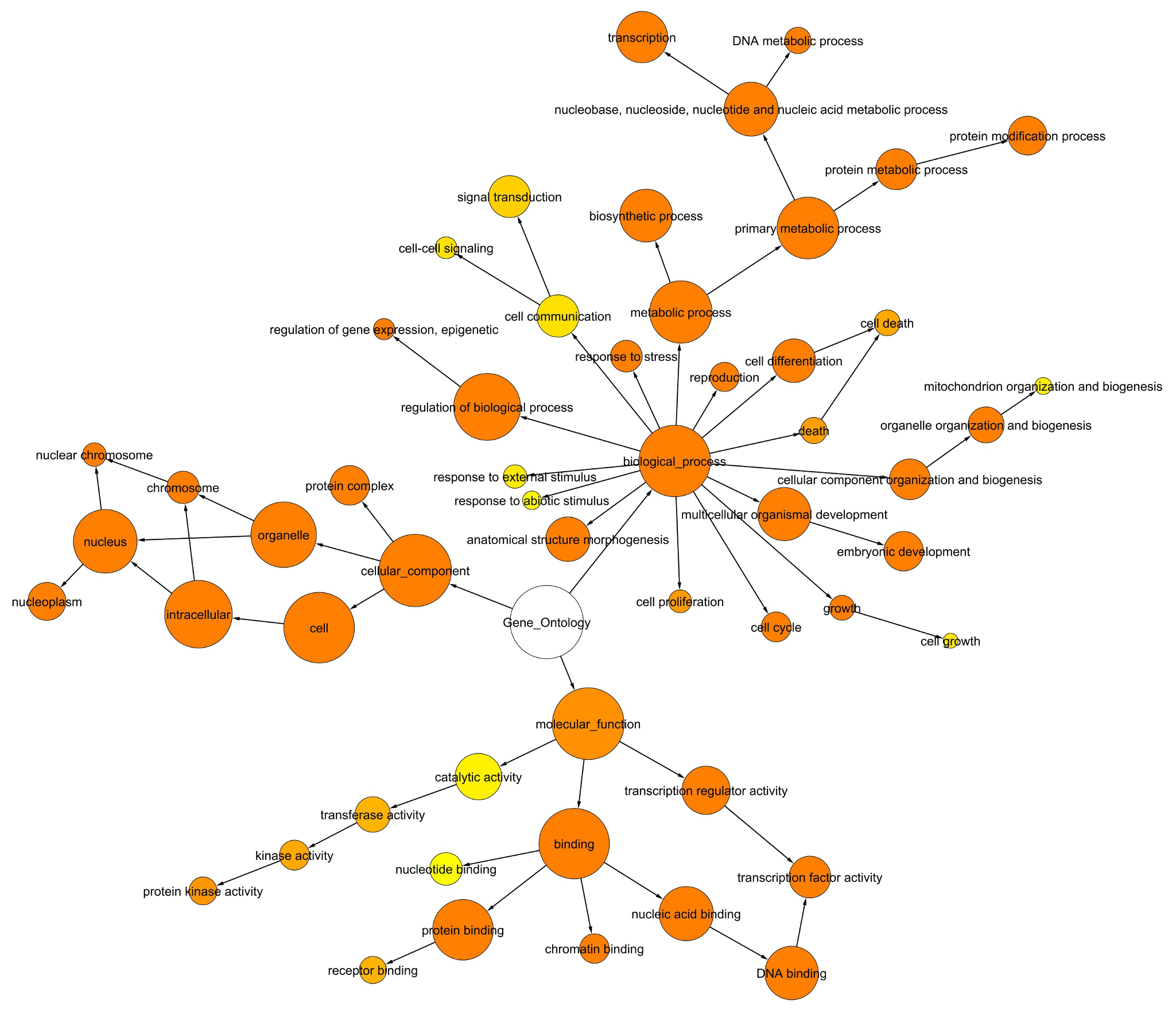


Figure 5. Result of GO analysis by BINGO.

In Figure 5, enriched cellular components are the nuclear chromosome and the nucleoplasm, reflecting the prominence of transcription factors and epigenetic factors in the *PluriNetWork*. Biological Process Terms are enriched for transcription, DNA metabolism, protein modification, cell differentiation / cell death, embryonic development, cell cycle, epigenetic regulation of gene expression and signal transduction. Enriched molecular functions such as transcription factor activity, chromatin binding, receptor binding and protein kinase activities fit well to the enriched biological processes.

***ExprEssence - Condensing Networks***

*ExprEssence* is a *Cytoscape* plugin analyzing a network of genes/proteins together with differential biological data, as measured in an experiment. It highlights the links across which the largest amount of change can be observed, given two experimental data sets. More specifically, *ExprEssence* condenses networks so that they contain only those links between genes/proteins, along which a large amount of change in (expression) values takes place. These links are called *most differentially altered.* The percentage of *most differentially altered* links to be highlighted can be set by the user, using a slider. Highlighting identifies hypotheses about the startup or the shutdown of interactions, stimulations and inhibitions.

**Installation**

The up-to-date *ExprEssence*.jar file can be downloaded at [sourceforge.net](http://sourceforge.net/projects/expressence/).

Move the file to the *plugins* folder in the main *Cytoscape* folder, and (re-)start *Cytoscape*.

**General *ExprEssence* Workflow**

The following is the general *ExprEssence* workflow.

1. Open *Cytoscape* and open a *Cytoscape* session file (a cys file such as [MEF_PIPS_IPS.cys](http://www.ibima.med.uni-rostock.de/IBIMA/PluriNetWork/MEF_piPS_iPS.cys)). If not already done, import your own gene expression data (or other high-throughput data, as described [here](http://cytoscape.wodaklab.org/wiki/Cytoscape_User_Manual/Attributes#Import_Attribute_Table_Files) and in [3]). Data are already imported for the case studies below. **In the Network tab of the Control Panel on the left, select the network you wish to condense.**
2. Click the *condense!* button in the tool bar (see Figure 6).


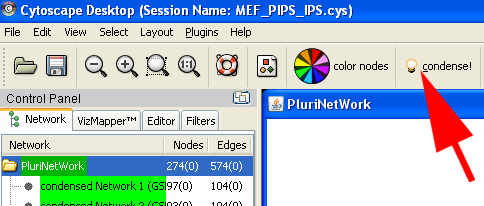


Figure 6. The condense button.

1. Choose the attribute-containing data for the first experiment on the left (such as GSE14012_MEF_mean; see Figure 7 below: arrow 1) and the attribute-containing data for the second experiment on the right (such as GSE14012_piPS_mean; see Figure 7 below: arrow 2).


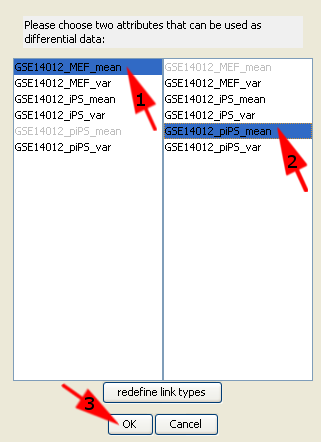


Figure 7. Window for selecting attributes in *ExprEssence*.

1. Press *OK* (Figure 7: arrow 3).
2. If you cannot provide variance data, click *No variance data* (see Figure 8).
   If you can provide variance data (such as *GSE14012_MEF_var* and *GSE14012_piPS_var*), select these as in step 3 and enter the number of replicates (here, for *GSE14012_MEF_var* the number is 4 and for *GSE14012_piPS_var* the number is 3, as in Figure 8). Then press *OK*.


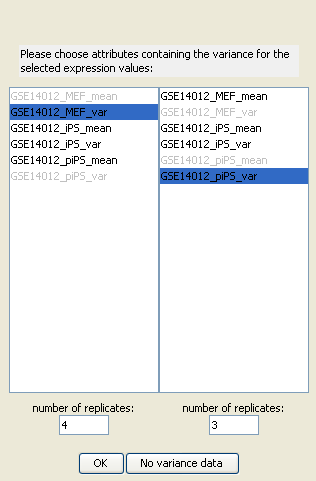


Figure 8. Window for selecting the variance attributes in *ExprEssence.*

1. A new network will then be generated and the Control Panel of *Cytoscape* (on the left side of the *Cytoscape* window) displays a slider to adjust the percentage *P* of *most differentially altered* links (see Figure 9), allowing to vary the degree of network condensation.


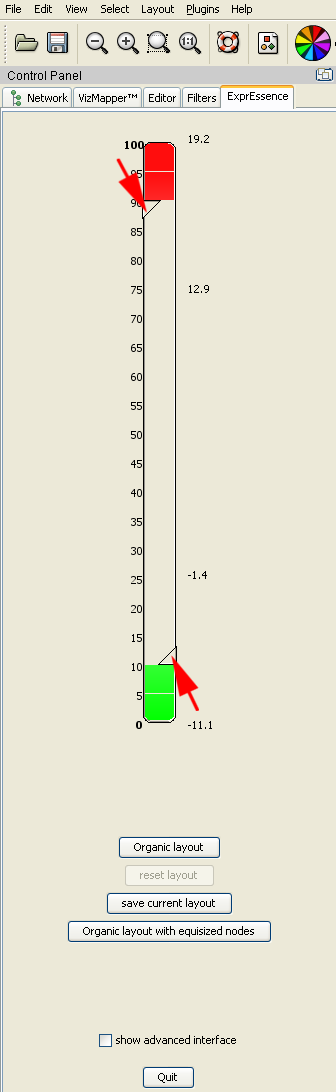


Figure 9. *ExprEssence* Slider.

The percentage *P* can be adjusted for both startups (red, top part of slider), and shutdowns (green, bottom part of slider). To keep the *P* % *most differentially altered* shutdowns, select a lower threshold of *P* %. To keep the *P* % *most differentially altered* startups, select an upper threshold of *100 – P* %.

In tabular form, an *ExprEssence* workflow asks you to provide the following data, with respect to the steps of the General *ExprEssence* Workflow:

| **Steps** | **To do** | **Example** |
| --- | --- | --- |
| File: (Step 1) | Select a Cytoscape file | [MEF_PIPS_IPS.cys](http://www.ibima.med.uni-rostock.de/IBIMA/PluriNetWork/MEF_piPS_iPS.cys) |
| Attribute: (Step 3)  mean values | Select mean values for the first (left) and the second (right) condition. | left: GSE14012_MEF_mean  right: GSE14012_piPS_mean |
| Attribute: (Step 4)  variances (replicates)  [optional] | Optional: Select variance data for the first (left) and the second (right) condition and provide the number of replicates. | left: GSE14012_MEF_var (replicates: 4)  right: GSE14012_piPS_var (replicates: 3) |
| Slider: (Step 6)  lower [optional] | Optional: Adjust the lower slider so that P % of the shutdowns are shown. | 10% |
| Slider: (Step 6)  upper [optional] | Optional: Adjust the upper slider so that 100-P % of the shutdowns are shown. | 90% |

Table 1. Basic *ExprEssence* user input using the *PluriNetWork*.

Finally, the following Table describes the general workflow of an *ExprEssence* analysis using the *PluriNetWork*:

| Input: | Gene expression measurements describing an experiment. We always compare two measurements, for example *before* and *after*, or *condition 1* and *condition 2.* |
| --- | --- |
| Analysis: | *ExprEssence* highlighting of the top startups and shutdowns of interactions, stimulations and inhibitions in the *PluriNetWork.* |
| Output: | Hypotheses for (regulatory) mechanisms acting in the course of the experiment, e.g. highlighting the *startup* of a *stimulation,* if the expression of *both stimulator and target go up*. |
| Interpretation: | Plausibility check / confirmation experiments for the (regulatory) mechanisms found. |

Table 2. General workflow of an *ExprEssence* analysis using the *PluriNetWork*.

**Case studies**

In the following case studies we used *ExprEssence,* combining the *PluriNetWork* with expression data and highlighting the most significant changes of gene expression along links connecting genes/proteins of the *PluriNetWork*.

**MEF to piPS and piPS to iPS**

**Transition from mouse embryonic fibroblast (MEF) to partially induced pluripotent stem cells (piPS) and from piPS to induced pluripotent stem cells (iPS)*.***

As described in the *PluriNetWork* paper, we used microarray data by Sridharan et al. [4] to investigate the induction of pluripotency.

| Input: | Microarray data describing (1) fibroblasts, (2) partially induced and (3) fully induced pluripotent stem cells. We first compare (1) and (2), and then (2) and (3). |
| --- | --- |
| Analysis: | *ExprEssence* highlighting of the top 10% startups and the top 10% shutdowns. |
| Output: | Hypotheses for (regulatory) mechanisms acting in the course of the induction of pluripotency. |
| Interpretation: | Comparison of our results with Sridharan et al [4]. |

Table 3. Workflow of the *ExprEssence* analysis of the induction of pluripotency.

The file [MEF_PIPS_IPS.cys](http://www.ibima.med.uni-rostock.de/IBIMA/PluriNetWork/MEF_piPS_iPS.cys) provides the *PluriNetWork,* gene expression data (plus variance data) mapped onto the *PluriNetWork,* as well as the already condensed networks, which we will interpret after describing how to produce them. Before integrating the gene expression data of Sridharan et al [4] (GSE14012), we normalized them (using the "rma" method from the affy package of Bioconductor [5]). As a result, nodes (genes) in the *PluriNetWork* now have the following attributes:

(1) GSE14012_MEF_mean and GSE14012_MEF_var: Mean and variance of gene expression in fibroblasts (MEF, mouse embryonic fibroblasts).

(2) GSE14012_piPS_mean and GSE14012_piPS_var: Mean and variance of gene expression in partially induced pluripotent stem cells (piPS).

(3) GSE14012_iPS_mean and GSE14012_iPS_var: Mean and variance of gene expression in induced pluripotent stem cells (iPS).

**Transition from MEF to piPS.**

*ExprEssence* is now used to derive the first condensed network comparing (1) MEF and (2) piPS. In the “General *ExprEssence* Workflow” [above](#GEW), the following data are provided, resulting in Figure 10 below:

| **Figure**  **Steps** | **10** |
| --- | --- |
| File: | [MEF_PIPS_IPS.cys](http://www.ibima.med.uni-rostock.de/IBIMA/PluriNetWork/MEF_piPS_iPS.cys) |
| Attribute:  mean values | left: GSE14012_MEF_mean  right: GSE14012_piPS_mean |
| Attribute:  variances (replicates) | left: GSE14012_MEF_var (replicates: 4)  right: GSE14012_piPS_var (replicates: 3) |
| Slider:  lower | 10% |
| Slider:  upper | 90% |

Table 4. User input for reproduction of Figure 10.


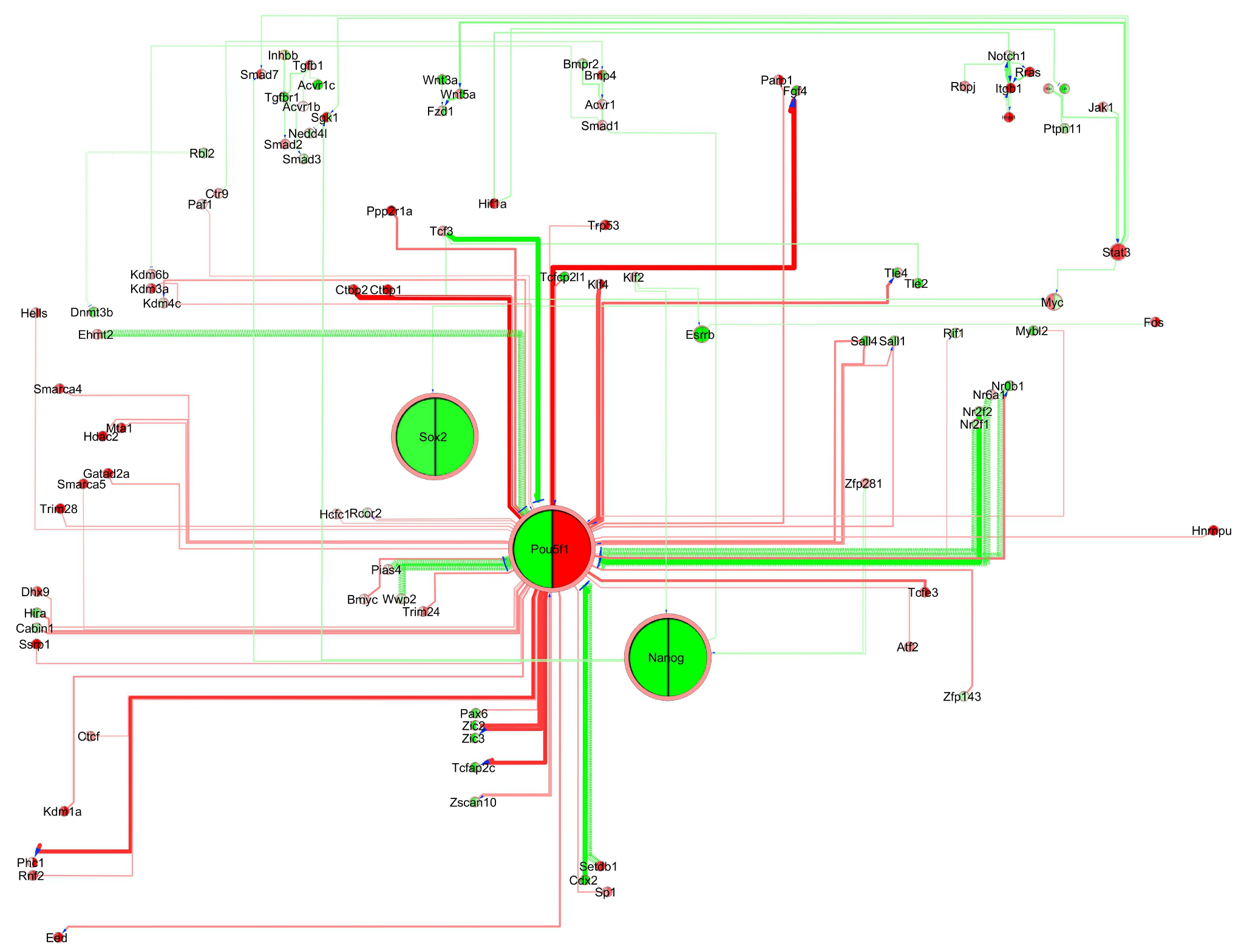


Figure 10. Transition from MEF to piPS.

To interpret the figure, we first describe some background on the experiment. According to Sridharan et al. [4], *partial induction* features changes associated with c-Myc, including metabolic regulation and transcriptional repression of somatic gene expression. Inspection of the *PluriNetWork* links to/from c-Myc indicates that its role in metabolic regulation is not documented well in the specific literature; Sridharan et al. [4] identify this feature by Gene Ontology analysis of target genes bound by c-Myc, based on ChIP-derived information. The role of c-Myc in transcriptional repression is concordant with the observation that in iPS generation, c-Myc may be substituted by small molecules with a histone deacetylation effect (e.g. valproic acid, VPA) [4],[6]. In the *PluriNetWork*, histone deacetylation and other epigenetic phenomena are included, but they are not connected to c-Myc. Moreover, we note that c-Myc is downregulated.

Instead, startup of the stimulation of epigenetic effectors is highlighted around Oct4/Pou5f1, based on two recent publications [7,8], owing to the strong upregulation of Oct4. Consequently, by *ExprEssence* condensation of the *PluriNetWork* highlighting putative mechanisms of *partial induction* (Figure 10), we find a pronounced startup of epigenetic phenomena, associated with the upregulation of Oct4/Pou5f1. In particular, Oct4 stimulation of the histone deacetylase Hdac2 starts up. Moreover, interactions of Oct4 with Hells, Smarca4, Mta1, Gata2a, Smarca5, Trim28, Dhx9, Hira, Cabin1, SSrp1, Kdm1a, Ctcf, Phc1, Rnf2, and Eed all start up. Also, Oct4/Pou5f1 inhibition by Cdx2 [9], by Wwp2 [10] and by some nuclear receptors (Nr2f1, Nr2f2, Nr6a1) is shut down. Around c-Myc, we observe a few shutdowns (of its stimulation by Stat3 and Tcf3, and of its Sox2 stimulation), owing to its downregulation from MEF to piPS cells.

**Transition from piPS to iPS.**

*ExprEssence* is now used to derive a second condensed network, comparing (2) piPS and (3) iPS. In the “General *ExprEssence* Workflow” [above](#GEW), the following data are provided, resulting in Figure 11 below:

| **Figure**  **Steps** | **11** |
| --- | --- |
| File: | [MEF_PIPS_IPS.cys](http://www.ibima.med.uni-rostock.de/IBIMA/PluriNetWork/MEF_piPS_iPS.cys) |
| Attribute:  mean values | left: GSE14012_piPS_mean  right: GSE14012_iPS_mean |
| Attribute:  variances (replicates) | left: GSE14012_piPS_var (replicates: 3)  right: GSE14012_iPS_var (replicates: 3) |
| Slider:  lower | 10% |
| Slider:  upper | 90% |

Table 5. User input for reproduction of Figure 11.

Sridharan et al. [4] note that the changes associated with Oct4, Sox2 and Klf4 *(full induction)* are skewed towards transcriptional regulation. Transcriptional regulation is represented well in the *PluriNetWork*, and we expect to find startups here. Sridharan et al. also hypothesize that Nanog may be a key factor for *full induction.*

*
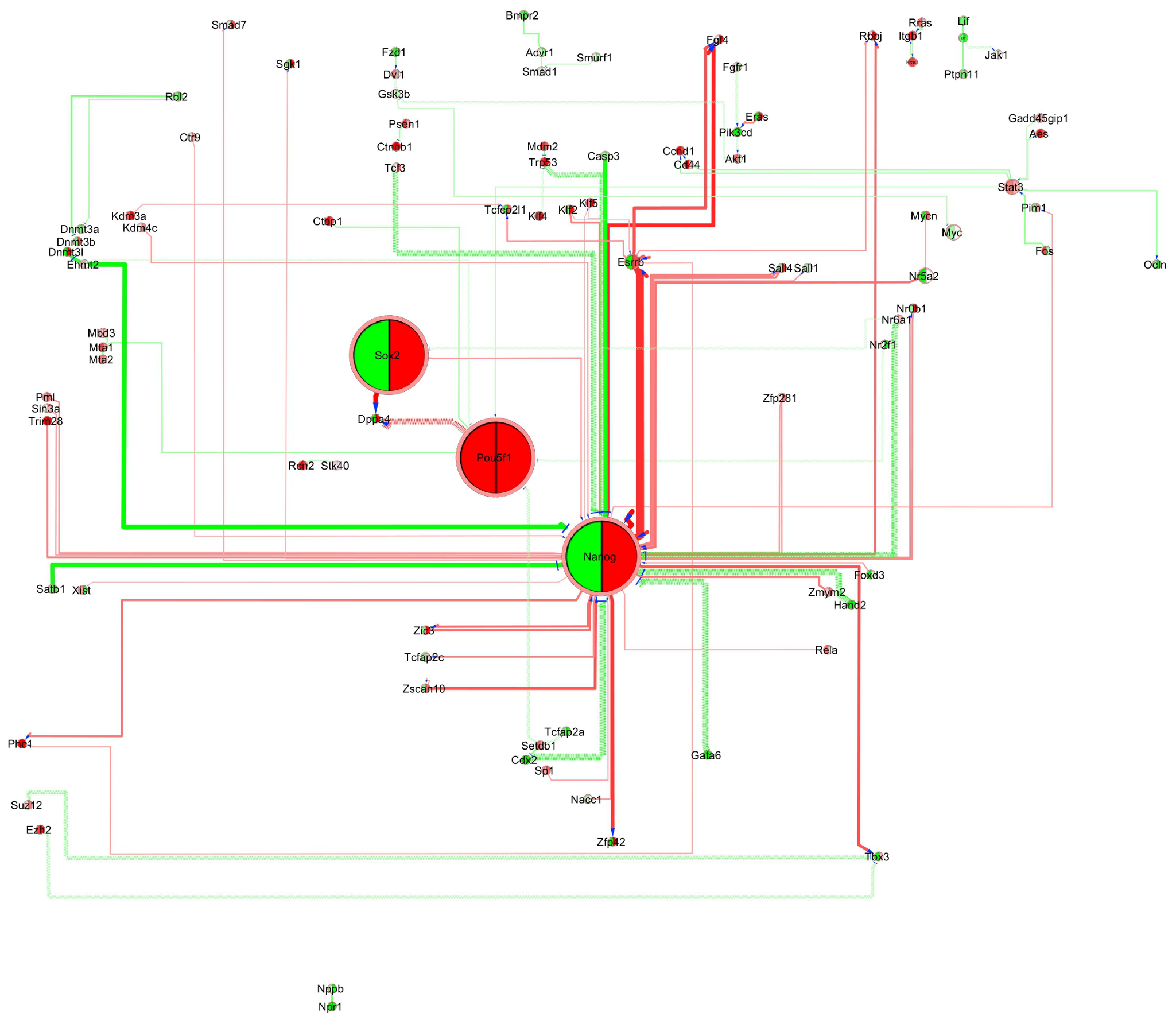
*

Figure 11. Transition form piPS to iPS.

Indeed, by *ExprEssence* condensation of the *PluriNetWork* highlighting putative mechanisms of *full induction* (Figure 11), we observe Nanog-driven startup of pluripotency-related transcription factors, such as Esrrb, Sall4, Tbx3, Zfp42 and Zic3. Sridharan et al. also report induction of 21 genes (Figure 4D in their paper). Inspecting their list, of the five genes included in the *PluriNetWork* (Tcfcp2l1, Lefty2, Tcl1, Dppa4 and Klf2), startup of stimulation of Tcfcp2l1, Dppa4 and Klf2 is highlighted in the condensed network (Figure 11). Startup is below-threshold in case of Lefty2, and Tcl1 is only connected to genes with negligible change in expression value; it is not connected to the main pluripotency factors. Also, inhibitions around Nanog are shut down, including inhibitions by Cdx2 (trophectoderm marker) and Gata6 (primitive endoderm marker). Coincidentally, *partial induction* involves shutdown of inhibition by Cdx2 only (see above), whereas *full induction* involves shutdown of inhibitions by both Cdx2 and Gata6, reflecting the embryonal developmental timeline. Finally, Sox2 stimulation of Dppa4 starts up, concordant with Sridharan et al. However, even though Sox2 changes about as much as Nanog in its expression level, this is the only highlighted effect around the Sox2 gene; most of the putative action happens around Nanog, concordant with Sridharan et al.

**Embryonic Stem Cell to Epiblast Stem Cell state**

Transition from embryonic to epiblast stem cell state.

As described in the *PluriNetWork* paper, we used microarray data by Greber et al. [11] (GSE10017) to investigate the transition from the embryonic stem cell state to the epiblast stem cell state.

| Input: | Microarray data describing four different conditions as described below (see [Table 9](#Table__9)). |
| --- | --- |
| Analysis: | *ExprEssence* highlighting of the top startups and shutdowns. |
| Output: | Hypotheses for (regulatory) mechanisms acting in the transition from the embryonic stem cell state to the epiblast stem cell state. |
| Interpretation: | Comparison of our results with the literature, and confirmatory experiments (see Figure 8 of the [*PluriNetWork* paper](http://www.ibima.med.uni-rostock.de/IBIMA/PluriNetWork/PluriNetWork.pdf)). |

Table 6. Workflow of the *ExprEssence* analysis of the ES / Epiblast transition.

*ExprEssence* is now used to derive the first condensed network, comparing (1) “12h PD LIF” and (2) “12h PD Jaki”. In the “General *ExprEssence* Workflow” [above](#GEW), the following data are provided, resulting in Figure 12B below; note that Figure 12 coincides with Figure 7 of the [*PluriNetWork* paper](http://www.ibima.med.uni-rostock.de/IBIMA/PluriNetWork/PluriNetWork.pdf):

| **Figure**  **Steps** | **12B** |
| --- | --- |
| File: | [Epiblast.cys](http://www.ibima.med.uni-rostock.de/IBIMA/PluriNetWork/Epiblast.cys) |
| Attribute:  mean values | left: 12h_PD+LIF_Signal  right: 12h_PD+JAKi_Signal |
| Attribute:  variances (replicates) | -  - |
| Slider:  lower | 5% |
| Slider:  upper | 95% |

Table 7. User input for reproduction of Figure 12B.

(The specific layout found in the figures below can be achieved by employing an *organic layout with nodes of equal size*, using the corresponding button in the *ExprEssence* tab, and manually moving the nodes.)


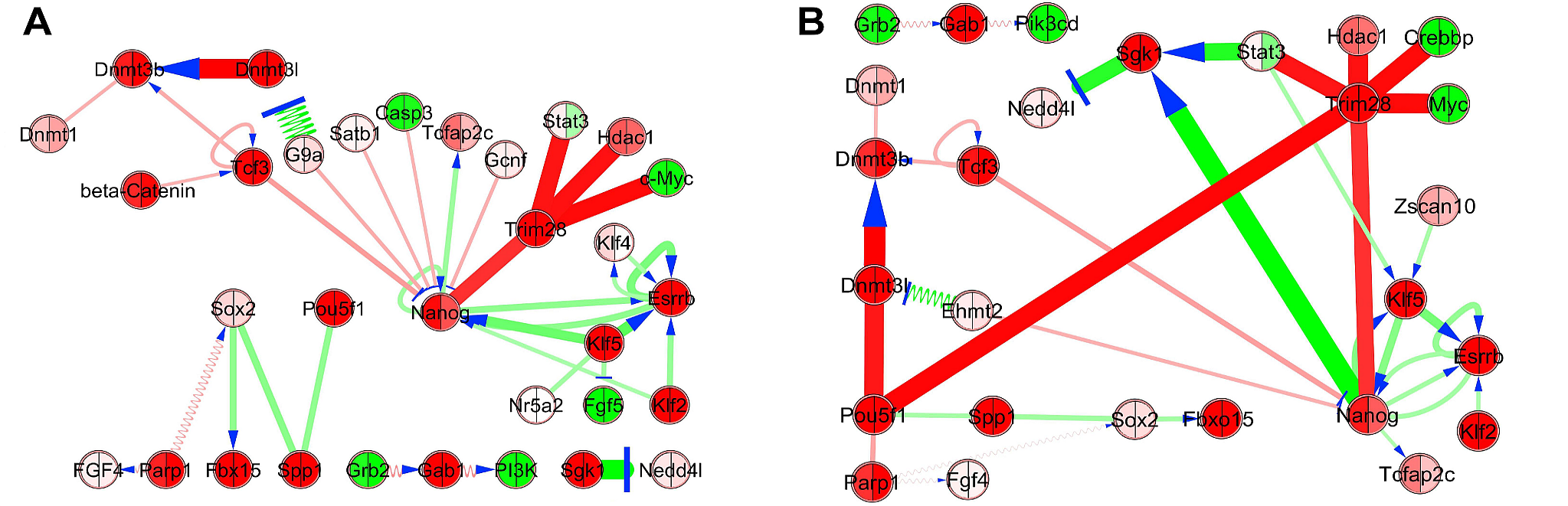


Figure 12. Condensed network based on comparing (1) “12h PD LIF” and (2) “12h PD Jaki”, based on an older version of the *PluriNetWork* (panel A), and the version described in the paper (panel B).

In the *PluriNetWork* paper, we compared the resulting condensed network (Figure 12B) to a network condensed with exactly the same microarray data, but with an older version of the PluriNetWork (Figure 12A, taken from [12]), and discussed some of the differences. Some more differences are apparent, and they are discussed here. In particular, as Klf4 is known to be upstream of Esrrb [13], we were puzzled that Klf4 is no longer featured in the condensed network based on the *PluriNetWork* we investigate here (Figure 12B). Moreover, we wondered why the presumed shutdown of Stat3-related signaling is not highlighted much stronger. Finally, we wondered why the shutdown of stimulation of Stat3 by Jak is not highlighted.

In Figure 12 (panels A and B), only the 5% most strongly differentially altered links of the PluriNetWork are displayed. For a threshold higher than 6.5%, the regulatory links between Klf4 and Esrrb appear, though (Figure 13A).


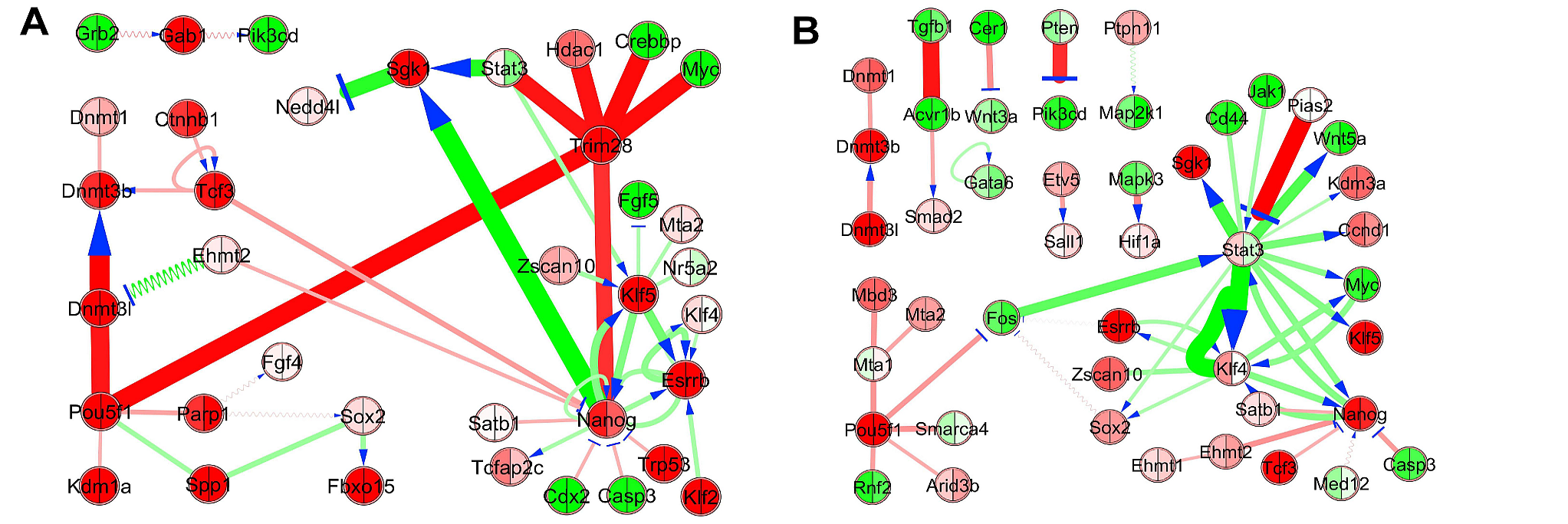


**Figure 13. Condensed network based on comparing conditions (1) “12h PD LIF” and (2) “12h PD Jaki”, using the original gene expression values (panel A) and log-transformed values (panel B).**

In the “General *ExprEssence* Workflow” [above](#GEW), the following data must be provided, resulting in Figure 13 above. (Figure 13B is based on log-transformed data, to be discussed below.)

| **Figure**  **Steps** | **13A** | **13B** |
| --- | --- | --- |
| File: | [Epiblast.cys](http://www.ibima.med.uni-rostock.de/IBIMA/PluriNetWork/Epiblast.cys) | [Epiblast.cys](http://www.ibima.med.uni-rostock.de/IBIMA/PluriNetWork/Epiblast.cys) |
| Attribute:  mean values | left: 12h_PD+LIF_Signal  right: 12h_PD+JAKi_Signal | left: X12h_PD.LIF_Signal  right: X12h_PD.JAKi_Signal |
| Attribute:  variances (replicates) | -  - | -  - |
| Slider:  lower | 6.5% | 6.5% |
| Slider:  upper | 93.5% | 93.5% |

Table 8. User input for reproduction of Figure 13.

In fact, while the *ExprEssence* link score measuring differential alteration of gene expression is only dependent on the change of the expression values of the two genes being linked, the addition of new nodes and links to the *PluriNetWork* modified the overall distribution of link scores so that the links between Klf4 and Esrrb are no longer below the 5% threshold, and they are only displayed if we visualize the 6.5% most differentially altered links. Closer inspection of Klf4, Klf2, Klf5, Esrrb and Nanog expression values and link scores (Table 9) reveals the reason underlying the fragility in observing Klf4 in our analysis of prominent mechanisms: In terms of the original value of the intensities in the data of GSE10017, Klf4 is expressed at a relatively low level, and its change (difference in intensities, not ratio of intensities) is low on this original scale.

| **Gene** | **12h PD LIF** | | **12h PD Jaki** | | **Link score to Esrrb** | |
| --- | --- | --- | --- | --- | --- | --- |
|  | **Absolute intensity value** | **Log-transformed intensity value** | **Absolute intensity value** | **Log-transformed intensity value** | **Absolute intensity value** | **Log-transformed intensity value** |
| Esrrb | 5173.35 | 12.34 | 4658.38 | 12.19 | -1029.93 | -0.30 |
| Klf2 | 2705.70 | 11.40 | 2379.04 | 11.22 | -841.62 | -0.34 |
| Klf4 | 306.38 | 8.26 | 90.90 | 6.51 | -730.44 | -1.90 |
| Klf5 | 2647.44 | 11.37 | 1893.18 | 10.89 | -1269.22 | -0.64 |
| Nanog | 1410.09 | 10.46 | 1022.71 | 10.00 | -902.34 | -0.61 |

Table 9. Selected absolute and log-transformed gene expression values and link scores for the ES to Epiblast transition. Absolute and log-transformed values of gene expression intensity for selected genes from GSE10017, for conditions (1) “12h PD LIF” and (2) “12h PD Jaki”, and corresponding link scores. The *ExprEssence* formula for the link score from a gene (Klf2, Klf4, Klf5, Nanog, Esrrb) to Esrrb takes the sum, over both genes, of the difference in gene expression between the two conditions under consideration (subtracting the value of condition “12h PD LIF” from that of condition “12h PD Jaki”). For example, in case of Klf4–Esrrb, we obtain for original values (90.90-306.38)+(4658.38-5173.35)= -730.45 and for log_2_-transformed values we calculate (6.51-8.26)+(12.19-12.34)= -1.9. For Esrrb, the link scores to itself are derived from its self-stimulation link.

The original scale highlights differential alterations in expression on any level, in this case focusing on the cooperation of Klf5, Klf2 and Nanog. Klf4 acts at a much lower concentration (assuming that concentration correlates well with gene expression), and its prominent role is thus revealed if we transform the data to a logarithmic scale (Figure 13B). On the logarithmic scale, the cooperation of the other factors at higher expression/concentration is no longer prominent in our case, and Klf4 takes center stage in downregulating Esrrb. We also observe that Stat3 is now highlighted much stronger as a hub of a large number of shutdowns, and this holds true to for Klf4 as well. The shutdown of Stat3 stimulation by Jak is now highlighted as well, though it does not receive a high link score. The reason for this is simply that our highlighting is based on differences in gene expression, but changes in pathway activity (here in case of the Jak/Stat pathway) do not necessarily imply changes in gene expression.

The question arises whether logarithmic or original intensity data shall be used for including gene expression data into networks such as the *PluriNetWork*. As demonstrated by [14], the logarithmic transformation may affect results on selecting differentially expressed genes. The study by Huang & Qu [15] states that without prior assumptions, there is no clear-cut recommendation, though. We believe that both kinds of data give complementary insight if network links are sorted by a measurement of importance such as the *ExprEssence* link score: the differential analysis of original data highlights changes across the full spectrum of intensities, and these can well be the cooperation of transcription factors all expressed on a medium level. Logarithmic data are better in the differential detection of small changes on a low expression level, often triggered by the high-affinity binding of a single transcription factor, exemplified by Klf4 and Stat3 in our example.

To further understand the relation of PluriNetWork data and expression data, we investigated two further conditions examined by [11]: (3) “12h FGF LIF” and (4) “12h FGF Jaki”, see Table 10.

| **Treatment condition** | **LIF** | **LIF inhibition by Jaki** |
| --- | --- | --- |
| *(effect on) downstream targets*  *(in italics)* | *Stat3*  *Klf4* | *Stat3*  *Klf4* |
| **FGF inhibition by PD** | (1) 12h PD LIF  ⇒ ES state maintained | (2) 12h PD Jaki  ⇒ partial transition to Epiblast |
| *MEK/ERK*  *Klf2* |
| **FGF** | (3) 12h FGF LIF  ⇒ partial transition to Epiblast | (4) 12h FGF Jaki  ⇒ transition to Epiblast |
| *MEK/ERK*  *Klf2* |

| **⊥** |
| --- |

| **⊥** |
| --- |

| **⊥** |
| --- |

| **⊥** |
| --- |

Table 10. Summary of the four different treatment conditions applied to mouse embryonic stem cells in [11].

In both cases, the FGF/MEK/ERK cascade was activated by supplementing the ES cell culture medium with FGF2 (and no inhibition of FGF/MEK/ERK signaling by PD (which is short for PD0325901) was done). Combined FGF/Jaki treatment in condition (4) promotes most effectively the transition to the epiblast state (indicated by dark grey background). As described, it is generally assumed that JAK inhibition triggers a transition signal via Stat3 and Klf4, while FGF stimulation shall de-repress Klf2 through activation of the MEK/ERK cascade. Thus, contrasting conditions (1) “12h PD LIF” and (2) “12h PD Jaki”, we observed a strong effect on Klf4 expression (Figure 13A and B). However, contrasting conditions (1) “12h PD LIF” and (3) “12h FGF LIF”, we would instead expect that ERK and Klf2 take center stage. In Figure 14A and B we find the most strongly differentially altered links for this scenario, using the absolute intensity and log-transformed data, respectively.


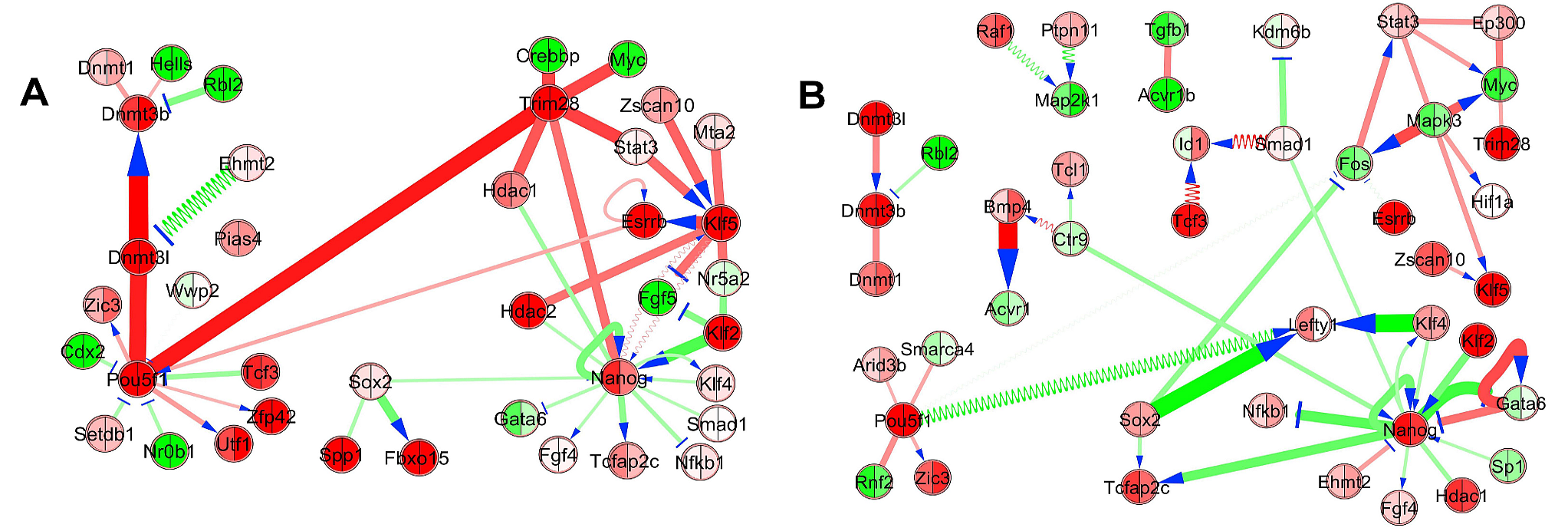


**Figure 14. Condensed network based on conditions (1) “12h PD LIF” and (3) “12h FGF LIF”, using the original gene expression values (panel A) and log-transformed values (panel B).**

In the “General *ExprEssence* Workflow” [above](#GEW), the following data are provided, resulting in Figure 14A and B above.

| **Figure**  **Steps** | **14A** | **14B** |
| --- | --- | --- |
| File: | [Epiblast.cys](http://www.ibima.med.uni-rostock.de/IBIMA/PluriNetWork/Epiblast.cys) | [Epiblast.cys](http://www.ibima.med.uni-rostock.de/IBIMA/PluriNetWork/Epiblast.cys) |
| Attribute:  mean values | left: 12h_PD+LIF_Signal  right: 12h_FGF2+LIF_Signal | left: X12h_PD.LIF_Signal  right: X12h_FGF2.LIF_Signal |
| Attribute:  variances (replicates) | -  - | -  - |
| Slider:  lower | 6.5% | 6.5% |
| Slider:  upper | 93.5% | 93.5% |

Table 11. User input for reproduction of Figure 14.

Using absolute intensities (Figure 14A), we indeed observe shutdown of three Klf2 links: its Nanog stimulation, Fgf5 inhibition and Nr5a2 (also known as LRH-1) interaction all go down. Interestingly, stimulations and inhibitions by and of Klf5 start up. The log-transformed data (Figure 14B) are highlighting a complementary hypothesis, namely the shutdown of Lefty1 activation, most prominently by Klf4 and Sox2. Lefty1 encodes an Activin antagonist. Since Activin signaling is known to be required for the maintenance of the epiblast cell state, this regulatory change might indicate a shift towards an epiblast-compatible expression of autocrine growth factors.

Finally, comparing condition (1) “12h PD LIF” to the “mixed case” of condition (4) “12h FGF Jaki”, displayed in Figure 15A and B, we find that both Klf2 and Klf4 (as well as Esrrb and Nanog) are at the center of shutdowns (original intensities, Figure 15A).


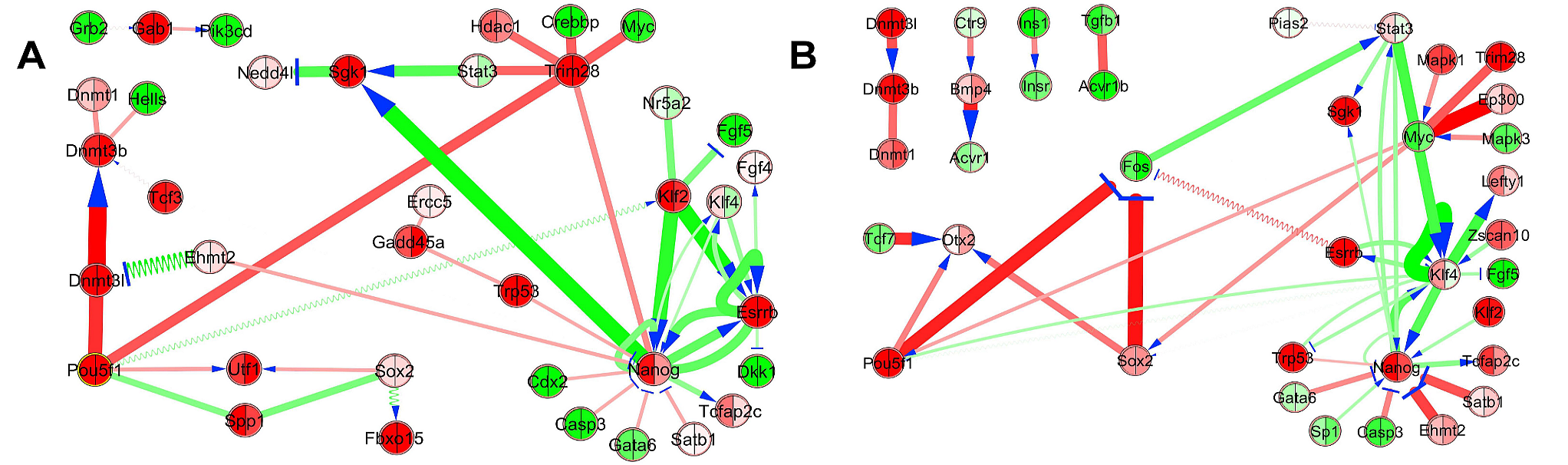


**Figure 15. Condensed network based on comparing conditions (1) “12h PD LIF” and (4) “12h FGF Jaki”, using the original gene expression values (panel A) and log-transformed values (panel B)**

In the “General *ExprEssence* Workflow” [above](#GEW), the following data are provided, resulting in Figure 15A and B above.

| **Figure**  **Steps** | **15A** | **15B** |
| --- | --- | --- |
| File: | [Epiblast.cys](http://www.ibima.med.uni-rostock.de/IBIMA/PluriNetWork/Epiblast.cys) | [Epiblast.cys](http://www.ibima.med.uni-rostock.de/IBIMA/PluriNetWork/Epiblast.cys) |
| Attribute:  mean values | left: 12h_PD+LIF_Signal  right: 12h_FGF2+Jaki_Signal | left: X12h_PD.LIF_Signal  right: X12h_FGF2.Jaki_Signal |
| Attribute:  variances (replicates) | -  - | -  - |
| Slider:  lower | 6.5% | 6.5% |
| Slider:  upper | 93.5% | 93.5% |

Table 12. User input for reproduction of Figure 15.

The complementary focus on small-intensity effects (logarithmic intensities, Figure 15A) highlights shutdowns around Klf4, which changes dramatically, but at a low absolute expression level. Moreover, we hypothesize a strong startup of inhibition of the protooncogene Fos. As in the case of inhibition of p53 [16], a pro-proliferative effect may be the result.

***NodeColor* – Visualisation of Gene Expression**

The *NodeColor* function of *ExprEssence* can be used to visualize gene expression values over many experiments for all genes in a network displayed using *Cytoscape*. You can select any number of attributes, where each attribute contains the expression values (floating point numbers) for a specific experimental condition. The default color scheme (green for low values, white for intermediate values, red for high values) can be adapted. As a result, each node is displayed using a pie-chart, where slices are color-coded based on gene expression (see Figure 16).

General workflow for the *NodeColor* function

1. Open *Cytoscape* and open a *Cytoscape* session file (cys file). If not already done, import your own high throughput data (as described [here](http://cytoscape.wodaklab.org/wiki/Cytoscape_User_Manual/Attributes#Import_Attribute_Table_Files)). In the *Network* tab of the *Control Panel* on the left, select the network for which you wish to visualize gene expression values.
2. Use the left mouse button to click on the *color nodes*-button in the toolbar.
3. Select the attribute(s) to visualize in the pie chart, by marking them and pushing the *+*-button.
4. Press *OK*.
5. Optional: Redefine the color scheme by changing the threshold values.
6. Press *OK*.
7. For all nodes in the network, the selected attributes will be displayed using pie-charts.


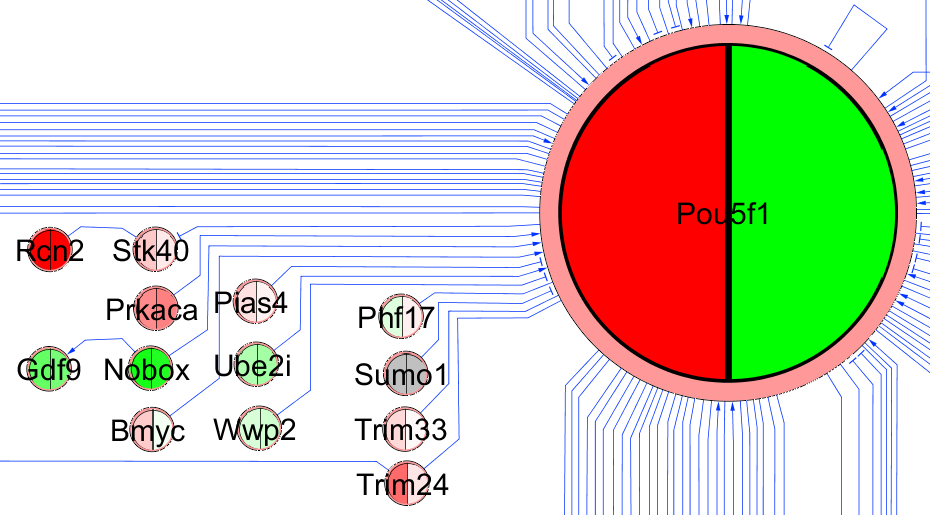


Figure 16. *NodeColor* visualization based on the data in [MEF_piPS_iPS.cys](http://www.ibima.med.uni-rostock.de/IBIMA/PluriNetWork/MEF_piPS_iPS.cys), displaying a part of the PluriNetWork (left: GSE14012_MEF_mean; right: GSE14012_piPS_mean).

**Visualization of Oct4 knockdown data using NodeColor.**

Visualizing the loss of pluripotency in the *PluriNetWork* should identify the agonists and antagonists of this cellular state. Taking the microarray data of [17], reported in [18] (GSE10477), we visualized change of mouse ES cell gene expression after two days of Oct4 (Pou5f1) conditional knockout. As described by [18], the cells begin to exhibit trophectodermal morphology after two days.

For visualization, we utilized the *NodeColor* function of *ExprEssence*. The NodeColor function is described in the [*ExprEssence* section](#NodeColorWorkflow).


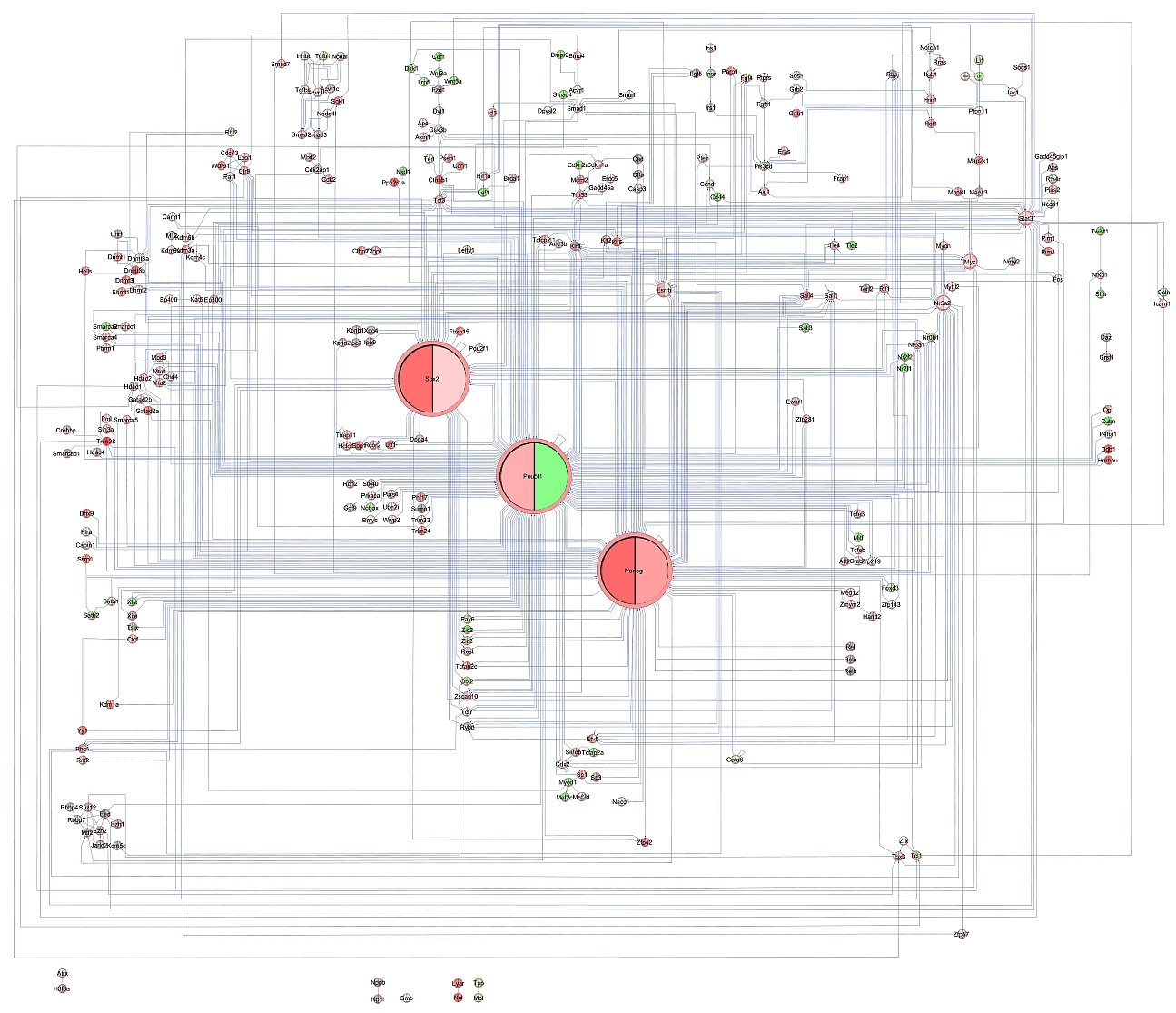


**Figure 17. Visualization of Oct4 knockdown data using NodeColor.**

Reproduction of Figure 17.

1. Use the Cytoscape file [Oct4_ko.cys](http://www.ibima.med.uni-rostock.de/IBIMA/PluriNetWork/Oct4_ko.cys).
2. Press the *color nodes* button.


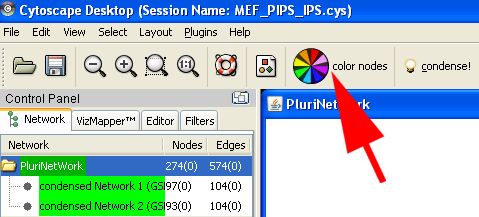


Figure 18. The *color nodes* button.

1. Select the attributes *GSE10573_Oct4_KO_d0_to_d1* and *GSE10573_Oct4_KO_d1_to_d2*. Press *OK*.
2. Use the *threshold values* low= 0, mediate= 8, high= 16. Press *OK*.

Expression values are visualized by coloring the nodes following the heatmap metaphor, mapping low values to green color, and high values to red color. Highly divergent values can then be identified easily by inspecting color difference. Intermediate levels of expression are represented by color of less intensity.

In the *PluriNetWork* of Figure 17, the coloring scheme allows us to observe an inverse correlation of expression values: agonists of pluripotency, including Oct4 (Pou5f1), Sox2, Nanog, Esrrb, Nr5a2 (also known as LRH-1), Klf2, Klf4, Klf5 and Fbxo15 are downregulated after two days. In contrast, antagonists of pluripotency, such as Cdx2, Tcfap2c, Gata6, Dkk1 (Wnt inhibitor), Cdkn1a & Fos (inhibited by Oct4), are more expressed after two days of Oct4 KO.

**References**

1. Ashburner M, Ball CA, Blake JA, Botstein D, Butler H, et al. (2000) Gene ontology: tool for the unification of biology. The Gene Ontology Consortium. Nat Genet 25: 25-29.

2. Camon E, Magrane M, Barrell D, Lee V, Dimmer E, et al. (2004) The Gene Ontology Annotation (GOA) Database: sharing knowledge in Uniprot with Gene Ontology. Nucleic Acids Res 32: D262-266.

3. Cline MS, Smoot M, Cerami E, Kuchinsky A, Landys N, et al. (2007) Integration of biological networks and gene expression data using Cytoscape. Nat Protoc 2: 2366-2382.

4. Sridharan R, Tchieu J, Mason MJ, Yachechko R, Kuoy E, et al. (2009) Role of the murine reprogramming factors in the induction of pluripotency. Cell 136: 364-377.

5. Irizarry RA, Hobbs B, Collin F, Beazer-Barclay YD, Antonellis KJ, et al. (2003) Exploration, normalization, and summaries of high density oligonucleotide array probe level data. Biostatistics 4: 249-264.

6. Huangfu D, Osafune K, Maehr R, Guo W, Eijkelenboom A, et al. (2008) Induction of pluripotent stem cells from primary human fibroblasts with only Oct4 and Sox2. Nat Biotechnol 26: 1269-1275.

7. Pardo M, Lang B, Yu L, Prosser H, Bradley A, et al. (2010) An expanded Oct4 interaction network: implications for stem cell biology, development, and disease. Cell Stem Cell 6: 382-395.

8. van den Berg DL, Snoek T, Mullin NP, Yates A, Bezstarosti K, et al. (2010) An Oct4-centered protein interaction network in embryonic stem cells. Cell Stem Cell 6: 369-381.

9. Niwa H, Toyooka Y, Shimosato D, Strumpf D, Takahashi K, et al. (2005) Interaction between Oct3/4 and Cdx2 determines trophectoderm differentiation. Cell 123: 917-929.

10. Liao B, Jin Y (2010) Wwp2 mediates Oct4 ubiquitination and its own auto-ubiquitination in a dosage-dependent manner. Cell Res 20: 332-344.

11. Greber B, Wu G, Bernemann C, Joo JY, Han DW, et al. (2010) Conserved and divergent roles of FGF signaling in mouse epiblast stem cells and human embryonic stem cells. Cell Stem Cell 6: 215-226.

12. Warsow G, Greber B, Falk S, Harder C, Siatkowski M, et al. ExprEssence – Revealing the essence of differential experimental data in the context of an interaction/regulation network. under review.

13. Jiang J, Chan YS, Loh YH, Cai J, Tong GQ, et al. (2008) A core Klf circuitry regulates self-renewal of embryonic stem cells. Nat Cell Biol 10: 353-360.

14. Li W, Suh YJ, Zhang J (2006) Does logarithm transformation of microarray data affect ranking order of differentially expressed genes? Conf Proc IEEE Eng Med Biol Soc Suppl: 6593-6596.

15. Huang S, Qu Y (2006) The loss in power when the test of differential expression is performed under a wrong scale. J Comput Biol 13: 786-797.

16. Krizhanovsky V, Lowe SW (2009) Stem cells: The promises and perils of p53. Nature 460: 1085-1086.

17. Niwa H, Miyazaki J, Smith AG (2000) Quantitative expression of Oct-3/4 defines differentiation, dedifferentiation or self-renewal of ES cells. Nat Genet 24: 372-376.

18. Endoh M, Endo TA, Endoh T, Fujimura Y, Ohara O, et al. (2008) Polycomb group proteins Ring1A/B are functionally linked to the core transcriptional regulatory circuitry to maintain ES cell identity. Development 135: 1513-1524.

**About**

Authors: Clemens Harder, Gregor Warsow, Georg Fuellen

Publications:

Contact: [fuellen@uni-rostock.de](mailto:fuellen@uni-rostock.de)
